# Association between Social Support and Depressive Symptoms in Informal Caregivers of Adult and Older Dependents: A Systematic Review and Meta-Analysis

**DOI:** 10.3390/jcm12206468

**Published:** 2023-10-11

**Authors:** Belén Gutiérrez-Sánchez, Vasiliki Orgeta, Catalina López-Martínez, Rafael del-Pino-Casado

**Affiliations:** 1 Department of Nursing, Faculty of Health Sciences, University of Jaén, 23700 Jaén, Spain; bgutierr@ujaen.es (B.G.-S.); rdelpino@ujaen.es (R.d.-P.-C.); 2 Division of Psychiatry, University College London, London W1T 7BN, UK; v.orgeta@ucl.ac.uk

**Keywords:** social support, depressive symptoms, informal caregivers, meta-analysis

## Abstract

Social support is an important determinant of a carer’s mental health. In recent decades, despite many studies reporting on the relationship between social support and depressive symptoms in informal caregivers of adult and older dependents, there are no systematic reviews synthesizing the available evidence. The purpose of the present study was to perform a systematic review and meta-analysis on the relationship between social support and depressive symptoms in informal caregivers of adults and older dependent people. We searched PubMed, CINAHL (EBSCO), PsycINFO (ProQuest), and Scopus, up to 15 January 2023 for studies. We applied no date or language limits to our search. A random-effects model was used to pool effect estimates. The included studies were also independently assessed for quality. Publication bias was evaluated by funnel plots, Egger’s regression test, and the Trim and Fill method. Ninety-three studies were included in the review, reporting on a total of 15,431 informal caregivers. We found a moderate negative association between perceived social support and caregiver depressive symptoms (78 studies; r = −0.35, 95% CI = −0.39, −0.31; low heterogeneity and low risk of publications bias) and a small negative association between received social support and caregiver self-reported depressive symptoms (12 studies; r = −0.14, 95% CI = −0.20, −0.07; low heterogeneity and low risk of publications bias). Our results indicate that social support is a clinically relevant construct for carer well-being and an important protective factor for depressive symptoms in informal caregivers of adult and older dependents.

## 1. Introduction

According to the World Health Organization (WHO), one in six people experience some form of disability, which represents 16% of the world’s population [1]. In Europe, over 135 million people currently live with some form of disability associated with a chronic disease that causes dependency, a figure that is expected to increase due to the increased longevity of populations [1].

In this context, the main source of care for people living with dependency is usually support provided by family members [2]. Informal care is defined as the support and care offered by relatives, friends, or other people providing unpaid care to dependents in their immediate social network [3]. Providing unpaid care has a series of negative consequences on the physical and psychological health of informal caregivers, as well as in the social and economic spheres. The most frequent consequences are experiencing emotional distress, higher levels of subjective caregiver overload, and clinically significant anxiety and depression [4]. It is currently estimated that depression is one of the most frequent adverse consequences of providing care, being present in more than 42% of informal caregivers [5], with rates up to 40.2% in informal carers of people surviving stroke [6] and 33.9% in carers of people living with Alzheimer’s disease [7].

Several factors have been found to be associated with the presence of depressive symptoms in family caregivers, including social support. Social support is considered to play an important role in maintaining a sense of well-being for carers [8] defined as “the existence or availability of people we can trust, people who let us know that they care about us, value us, and love us” [8]. Social support can positively influence the physical and mental health of informal caregivers, contributing as a protector or buffer against stressors. It has been classified according to its purpose into specific dimensions: emotional (emotional support), instrumental (task help), and informational (information) [8]. Another classification evident in the literature is that between perceived or received social support. The first relates to the assessment of the availability of support when needed and its adequacy and/or quality, while received support is associated with the nature and frequency of specific support transactions [8].

Several theoretical models have been put forward to explain the effects of social support on mental health outcomes [9,10]. One of these includes the stress and coping models derived from the transactional stress theory of Lazarus and Folkman [9]. In this theory, stress is defined as the result of the interaction between the person and their environment, where when the latter is perceived as threatening or overwhelming in relation to the resources available to the individual, it can endanger well-being. Transactional stress theory defines social support as a variable that influences the stress appraisal process thereby directly or indirectly influencing the experience of stress; as a result, access to or lack of social support in specific but similar situations can differentially impact individuals [7]. In line with these models, Cohen and Wills [10] have argued that social support could play a key role in how individuals perceive stress, with provision to and access to certain resources making individuals perceive a current situation as less stressful.

Several empirical studies have shown that social support may have a protective effect on the onset of depressive symptoms in informal caregivers of dependent adults and older people [11,12,13]. However, the level of evidence of this protective effect remains unclear [14], with no systematic reviews consistently analysing the relationship between social support and depressive symptoms in informal caregivers. This may have important implications for carers’ emotional health, as it remains unknown which types of social support interventions may be most effective in promoting positive mental health outcomes for carers. There is also currently limited knowledge on how specific factors such as type of social support (perceived vs. received), cause of caregiving dependency, and methodological quality of studies affect the association between social support and carer depressive symptoms.

Therefore, the purpose of this review was to systematically summarize and synthesize the evidence by providing an average effect estimate of the relationship between social support and depressive symptoms in informal caregivers of dependent adults and older people. The secondary objective was to rate the quality of the evidence. The research questions that guided this systematic review were:

- Is high perceived social support associated with fewer depressive symptoms?
- Is high received social support associated with fewer depressive symptoms?

## 2. Materials and Methods

### 2.1. Design

A quantitative systematic review with meta-analysis was conducted following the recommendations of PRISMA [15] and the Cochrane Handbook [16] and registered with PROSPERO [17] (id: CRD42023405918).

### 2.2. Search Strategy

We carried out a systematic search up to 15 January 2023 in the following databases: PubMed, CINAHL, PsycINFO, and Scopus; details of the search strategy are presented in Table 1. No time or language filters were used (Table S1). For example, the search string used in PubMed was: ((Depression[mj] OR ((Depress*[tiab]) NOT MEDLINE[sb])) AND (Caregivers[mj] OR ((Caregiv*[tiab] OR Care giv*[tiab] OR Carer*[tiab]) NOT MEDLINE[sb])) AND (Social support[mh:noexp] OR ((social network[tiab] OR informal support[tiab] OR received support[tiab] OR perceived support[tiab]) NOT MEDLINE[sb]))) NOT (clinical trial[pt] OR randomized clinical trial[tiab] OR randomized controlled trial[tiab] OR qualitative study OR qualitative research).

### 2.3. Eligibility Criteria

To carry out the selection of studies, the following inclusion criteria were established: (1) original studies, (2) reporting on the relationship between social support and depressive symptoms, (3) in informal caregivers aged 18 years of age or over, (4) of adult and older dependents, (5) that presented adequate statistical data to assess the magnitude of the association or size of the effect. 

### 2.4. Data Extraction and Synthesis

Two review authors (BGS and RdPC) extracted data independently in a standardised way. This included: the first author and year of publication, sample (type of sampling and sample size), type of design, type of social support (perceived or received, both global and separate dimensions), type of social support scale used, depressive symptoms scale, chronic condition of the care recipient and size of the association or effect. The measures of association or effect used were the correlation coefficient or another measure that could be transformed into a correlation coefficient. The conversion of other effect measures to correlation coefficients was carried out by the statistical software used.

### 2.5. Ratings of Quality Assessment

For the evaluation of the methodological quality of the included studies, we followed the recommendations of the Cochrane Handbook in regards to rating specific areas of quality as opposed to using overall scores for each study. Thus, we carried out the evaluation of methodological quality by assessing selection, classification, and confounding biases based on criteria proposed by Viswanathan et al. [18] and Boyle [19] which were: (1) type of sampling (use of probability sampling or not; selection bias); (2) validity and reliability of measurements used (content validity and internal consistency of the questionnaires in the target population or similar; classification bias); this criterion was mandatory for a study to be included in the meta-analysis; (3) control for confounding (control for at least one measure of caregiver objective burden) and (4) for longitudinal studies, attrition rate (follow-up rate of 80% of the original population participating in the study; selection bias).

With respect to confounders, objective burden was considered necessary due to its association with depressive symptoms [20], and included functional ability, cognitive impairment, and behavioural problems [21]. Because these measures are interrelated [22], we considered as adequate studies controlling for at least one of the previous measures of objective burden. When statistical adjustment was performed, we considered confounding bias to be absent if the variation in the point estimate was less than 10%. Two review authors independently assessed study quality (BGS and RdPC).

### 2.6. Certainty Assessment

Based on the recommendations of the Grading of Recommendations Assessment Development and Evaluation (GRADE) guidelines [23], we assessed the quality of evidence by rating the methodological quality of the included studies (see previous paragraph), which included inconsistency (heterogeneity), imprecision and publication bias. Inconsistency refers to the variation of the effect estimates having excluded the main causes of this variation (e.g., sampling bias), allowing us to investigate heterogeneity. Imprecision allows us to study the effects of sample size, through the amplitude of confidence intervals, sample size, and number of events. Publication bias enabled us to assess whether there is a high probability of unreported studies, mainly due to the absence of effects, or not including all relevant outcome variables.

Assessment of inconsistency and publication bias are described below. We assessed imprecision by evaluating the number of studies included in each meta-analysis (small: <5 studies, medium: 5–10 studies, and adequate: >10 studies) and the average sample size (low: <100 participants, intermediate: 100–300 participants, and high: >300 participants) [24].

### 2.7. Analyses

A random-effects model was used in the meta-analysis to allow generalization of the findings to any caregiving population of adult and older dependents, as recommended by Cooper et al. [25].

For the heterogeneity analysis, the Q-test was used, alongside the degree of inconsistency (I^2^) of Higgins et al. [26]. The Q-test indicates heterogeneity when the *p*-value is greater than 0.1. The degree of inconsistency (I^2^) is the proportion of the variability observed in the effect of the intervention (between studies) that is due to heterogeneity between studies and not to chance. It comprises values between 0% (no heterogeneity) and 100% (maximum heterogeneity), with values of 25% indicative of little heterogeneity; 50% as moderate, and 75% as high [26]. Following the recommendations of Guyatt et al. [27], we used several methods to assess publication bias. These methods were funnel plot evaluation, the Egger’s test [28], and the Trim and Fill method [29]. The Egger test is the regression of the funnel plot measuring whether the slope of the regression is equal (there is no publication bias) or different (there is) from 0, with a *p*-value greater than 0.1 indicative of a low risk of publication bias [28]. Following the recommendations of the Cochrane Handbook [16], the Egger test was only assessed in meta-analyses with at least 10 included studies. The Trim and Fill method calculates the estimated effect after correcting for possible asymmetry in the funnel plot by eliminating small studies that cause asymmetry and imputing the missing studies necessary [29]. Thus, by comparing the value of the combined effect with that estimated by the Trim and Fill method, we can estimate whether there is no publication bias (previous values are the same) or if there is an influence on the combined effect.

We conducted a sensitivity analysis to investigate the robustness of our findings. We used the leave-one-out method by eliminating one study at a time and analysing the remaining k-1 studies each time. Subgroup analyses were performed to analyse the robustness of our findings and the possible effect of moderators on the combined effect estimate. We selected the following moderators: type of study design (cross-sectional vs. longitudinal), quality criteria (selection bias, classification bias, and confounding), and chronic condition of the care recipient (frail older people, dementia, cancer, mental health disorder, and stroke). In addition, we conducted meta-regressions to analyse the possible moderating effect of caregiver age (mean) and caregiver gender (% female).

All analyses were performed using the Comprehensive Meta-analysis program 3.3 (Biostat, Englewood, NJ, USA).

**Table 1:** Description of studies included in the review.

Study (Author-Year) Country	N	Mean Age (SD) and Range	Percentage of Female (%)	Design	Recipients of Care	Type of Social Support	Measure of Social Support **	Measuring Depressive Symptoms **
Aggar 2010 [30] Australia	93	65.8 (13.6) 37–95	59.1	Cross-sectional	Frail older people	Perceived (global)	CRA	HADS
Ali 2016 [31] Pakistan	90	34.9 (8.9) 20–45	84.4	Cross-sectional	Stroke	Perceived (global)	MSPSS	DASS-42
Amorin 2009 [32] Portugal	46	35 (N/A) N/A	80.4	Cross-sectional	Cancer	Perceived (emotional)	ESSS	EADS-21
Ar 2017 [33] Turkey	190	51.4 (8.7) 26–77	89.5	Cross-sectional	Dementia	Perceived (global)	MSPSS	BDI
Arevalo-Flechas 2014 [34] United States	202	64.7(8.9) 47–83 *	76.4	Cross-sectional	Dementia	Perceived (global)	PRQ-85	HADS
Asti 2006 [35] Turkey	130	43.9 (8.52) 27–61 *	81.5	Cross-sectional	Dialysis	Perceived (global)	PSS	BDI
Au 2009 [36] China	134	54.5 (13.2) 28–81	74.6	Cross-sectional	Dementia	Perceived (global, emotional, and instrumental)	MSSS	CES-D
Azevedo 2017 [37] Brazil	115	68 (N/A) 59–76	64.3	Cross-sectional	Palliative care	Perceived (global)	MOS-SS	CES-D
Baillie 1988 [38] United States	87	52.5 (13.9) 22–91	76	Cross-sectional	Frail older people	Perceived (emotional)	Ad hoc	POMS
Ballard 1995 [39] United Kingdom	109	64.3 (13.5) 37–91 *	80.7	Cross-sectional	Dementia	Perceived (global)	Ad hoc	Cornell
Bambara 2014 [40] United States	42	51.6 (9.8) 32–71 *	90.5	Cross-sectional	Multiple sclerosis	Perceived (global)	SSSI	PHQ-9
Bergman 1992 [41] United States	94	70.2 (8.9) 52–88 *	69.2	Cross-sectional	Dementia	Perceived (global)	PRQ-85	CES-D
Biggati 2011 [42] United States	78	51.2 (12.6) 26–76 *	0	Cross-sectional	Cancer	Perceived (global)	ISEL	CES-D
Bonsu 2019 [43] Africa	100	33.2 (8.9) 15–51 *	79	Cross-sectional	Severe burns injuries	Perceived (global)	MSPSS	BDI
Burgeois 1996 [44] United States	100	71.9 (7.3) 57–87 *	55	Cross-sectional	Dementia	Perceived (global)	ISEL	CES-D
Burton 2008 [45] United States	50	72.4 (10.2) 52–93 *	80	Cross-sectional	Palliative care	Perceived (global)	Ad hoc	CES-D
Butler 2001 [46] United States	62	58 (N/A) 31–81	75.8	Cross-sectional	Frail older people	Perceived (global)	Ad hoc	CES-D
Cabral 2014 [47] Portugal	104	52 (N/A) 22–77	62.5	Cross-sectional	Mental health	Perceived (global)	ESSS	EADS-21
Calvete 2011 [48] Spain	223	49.9 (12.6) 20–77	72.2	Cross-sectional	Traumatic brain injury	Perceived (global, emotional, instrumental)	FNQ	CES-D
Cardenas 2014 [49] United States	264	57.5 (13) 21.5–84 *	100	Cross-sectional	Dementia	Perceived (global)	ISSB	CES-D
Chai 2018 [50] Asia	165	45 (14.6) 16–74 *	54.4	Cross-sectional	Mental health	Perceived (global)	MSPSS	QIDS-SR 16
Chou 2010 [51] China	350	66.6 (7.7) 55–87	44.9	Cross-sectional	Intellectual disability	Perceived (global)	SSS	CES-D
Chow 2012 [52] China	158	75.6 (6.8) 55–90	61.4	Cross-sectional	Frail older people	Perceived (global)	SSSQ	GDS
Clyburn 2000 [53] Canada	613	58.8 (13.5) 32–86 *	71	Cross-sectional	Dementia	Received (global)	Ad hoc	CES-D
Crespo 2005 [54] Spain	108	57.2 (11.5) 34–78 *	82.2	Cross-sectional	Frail older people	Received and perceived (global)	SSSQ	BDI
Cumming 2008 [55] Australia	116	66.9 (13.3) 32–92	71	Cross-sectional	Stroke	Perceived (global)	MOS-SS	IDA Scale
Decker 1989 [56] United States	67	55.9 (N/A) 17–75	88	Cross-sectional	Spinal cord injury	Perceived (global)	Ad hoc	CES-D
Del Pino Casado 2022 [57] Spain	81	57.6 (12.5) 28–89	87.3	Longitudinal (repeated measures)	Frail older people	Perceived (global)	Duke-UNC	Goldberg
Durkin 2010 [58] United States	130	63.4 (15) 20–87	84	Longitudinal (repeated measures)	Frail older people	Perceived (global)	ISEL	CES-D
Faber 2005 [59] United States	310	38.9 (7.3) 22–62	100	Cross-sectional	Cancer	Perceived (global)	ISEL	CES-D
Gibson 2013 [60] United States	1218	62.2 (13.2) 36–89 *	82	Cross-sectional	Dementia	Received (global)	K & B-C	CES-D
Giovannetti 2015 [61] Italy	129	52.8 (13.1) 27–79*	68.2	Cross-sectional	Disorders of consciousness	Perceived (global)	MOS-SS	BDI-II
Grant 2000 [62] United States	52	53.7 (16) 22–81	82.7	Cross-sectional	Stroke	Perceived (global, emotional, instrumental)	ISEL	CES-D
Grant 2001 [63] United States	40	53.3 (N/A) 22–81	85	Cross-sectional	Stroke	Perceived (global)	ISEL	CES-D
Graven 2020 [64] United States	530	41.4 (10.4) 21–62 *	49.1	Cross-sectional	Heart failure	Perceived (global)	ISEL	CES-D
Haley 1987 [65] United States	54	56.1 (16.3) 20–87	80	Cross-sectional	Dementia	Perceived (global)	HDLS	BDI
Han 2014 [66] China	301	46.7 (14) 19–75 *	63	Cross-sectional	Cancer	Perceived (global)	MSPSS	CESD-10
Harwood 2000 [67] United States	64	63.8 (14.9) 27–90	70	Cross-sectional	Dementia	Perceived (global)	PESS	CES-D
Hasson-Ohayon 2010 [68] Israel	150	56.2 (11) 34–78 *	100	Cross-sectional	Cancer	Received (global)	CPASS	BSI
Hobbs 1997 [69] United States	100	65.6 (8.12) 55–86	100	Cross-sectional	Mental health	Perceived (global)	PESS	CES-D
Hwang 2011 [70] United States	35	51.7 (12.9) 18–71	60	Cross-sectional	Pulmonary hypertension	Perceived (global)	MOS-SS	PHQ-9
Jeong 2017 [71] Korea	39	45 (12.8) 19–71 *	72.2	Cross-sectional	Cancer	Perceived (global)	Duke-UNC	HADS
Khusaifan 2017 [72] Saudi Arabia	122	N/A	78.7	Cross-sectional	Dementia	Perceived (global)	MSPSS	HDRS
Kiral 2017 [73] Turkey	141	59.7 (12.7) 32–85 *	77	Cross-sectional	Dementia	Perceived (global)	MSPSS	BDI
Koerner 2010 [74] United States	61	56.7(13.2) 30–83 *	73	Cross-sectional	Frail older people	Perceived (global)	Walen & Lanchman	HSC
Kruithof 2016 [75] Netherlands	183	62.5 (10.9) 41–84 *	78.7	Longitudinal (repeated measures)	Stroke	Received (global)	SSL-12-I	HADS
Kusku 2009 [76] Turkey	51	42.2 (11.1) 20–64 *	84.3	Cross-sectional	Cancer	Perceived (global)	MSPSS	BDI
Lakey 2002 [77] United States	100	49 (N/A) N/A	100	Cross-sectional	Dementia	Perceived (emotional)	QRI	CES-D
Lee 2003 [78] China	69	53 (14.4) 23–82	84	Cross-sectional	Dementia	Perceived (global)	PRQ-85	CES-D
Leibach 2013 [79] United States	81	43.4 (15.3) 13–74 *	66.7	Cross-sectional	Multiple sclerosis	Perceived (global)	ISEL	PHQ-9
Li 1997 [80] United States	252	65.4 (8.3) 49–82 *	100	Cross-sectional	Frail older people	Received (global, emotional, instrumental)	Ad hoc	CES-D
Li 2019 [81] China	557	57 (6.7) 44–70 *	47.2	Cross-sectional	Frail older people	Perceived (global)	MSPSS	CES-D
Losada 2010 [82] Spain	334	58.6 (12.9) 28–85	77.8	Cross-sectional	Dementia	Received (global)	PSQ	CES-D
Luchsinger 2015 [83] United States	139	59.3 (10.4) 39–80 *	85.7	Cross-sectional	Dementia	Perceived (global)	SSNL	GDS
Majerovitz 2007 [84] United States	103	56 (N/A) N/A	74	Cross-sectional	People living in nursing homes	Perceived (global)	SSSQ	CES-D
MaloneBeach 1995 [85] United States	57	58.3 (11.1) 22–83	100	Cross-sectional	Dementia	Received (global, emotional, instrumental)	Ad hoc	CES-D
Manso Martínez 2013 [86] Spain	88	56.6 (12.2) 32–81 *	84.1	Cross-sectional	Frail older people	Perceived (global)	Duke-UNC	HADS
Moral Serrano 2003 [87] Spain	215	55.3 (14.6) 26–85 *	87	Cross-sectional	Older people hospitalized at home	Perceived (global)	Duke-UNC	Goldberg
Neri 2012 [88] Brazil	176	71.8 (4.9) 68–90	70.7	Cross-sectional	Frail older people	Perceived (global)	ISEL	GDS
Nuwamanya 2023 [11] Africa	336	39.2 (11.5) 16–62 *	60.4	Cross-sectional	Cancer	Perceived (global)	MSPSS	PHQ-9
Pagel 1987 [89] United States	68	65 (9) 35–85	63.2	Cross-sectional	Dementia	Perceived (global)	GSS	BDI
Pagnini 2010 [90] Italy	40	55.6 (12.3) 51–80 *	70	Cross-sectional	Amyotrophic lateral sclerosis	Perceived (global)	MG-SS	BDI-II
Pearce 2006 [91] United States	162	51 (13.3) 24–78 *	73	Cross-sectional	Terminal cancer	Perceived (global)	ISEL	DSM-IV
Raad 2020 [14] United States	558	46.1 (14.1) 18–74 *	58	Cross-sectional	Traumatic brain injury	Perceived (global)	TBI-CareQOL	TBI-CareQOL
Rapp 1998 [92] United States	65	61.3 (14.4) 33–90 *	76.8	Cross-sectional	Dementia	Perceived (global)	MOS-SS	CES-D
Rauktis 1995 [93] United States	106	59 (N/A) 30–84	86	Cross-sectional	Mental health	Perceived (global)	PSI	CES-D
Rivera 1991 [94] Africa	165	58.6 (10.9) 30–85	100	Cross-sectional	Frail older people	Perceived (global)	ASSIS	SADS
Riverra–Navarro 2018 [95] Spain	326	60.1 (14.5) 31–89 *	67.2	Cross-sectional	Dementia	Perceived (global)	Duke-UNC	HADS
Robinson 1989 [96] United States	78	65 (N/A) 47–85	100	Cross-sectional	Dementia	Received (global)	ISSB	CES-D
Robinson 1994 [97] United States	40	65 (N/A) 52–80	100	Cross-sectional	Dementia	Perceived and Received (global)	GSS/SNL	CES-D
Rodakowski 2013 [98] United States	173	53 (15) 23–83 *	76	Cross-sectional	Spinal cord injury	Received	REACH trial	CES-D
Rodi 2015 [99] United States, United Kingdom and Australia	87	N/A (N/A) 18–74	86.2	Cross-sectional	Cancer	Perceived (global)	MOS-SS	BDI-II
Sahin 2012 [100] Turkey	60	54.7 (N/A) N/A	18	Cross-sectional	Cancer	Perceived (global)	PSS-Fa	BDI
Sandoval 2019 [101] Chile	377	51.7 (15.4) 15–87	85.1	Cross-sectional	Frail older people	Perceived (global)	Duke-UNC	CES-D
Schulz 1991 [102] United States	172	57.8 (N/A) N/A	18	Repeated measures with cross-sectional correlations	Dementia	Perceived (global)	ISEL	CES-D
Schumacher 1993 [103] United States	75	43.8 (14.7) 18–75	51	Cross-sectional	Cancer	Perceived (global)	Ad hoc	POMS
Schwarz 2000 [104] United States	100	64.7 (13.4) 29–88	74	Repeated measures with cross-sectional correlations	Frail older people	Received (global)	MISSB	CES-D
Scicolone 2018 [105] United States	249	64.3 (11.1) 30–89	92.5	Cross-sectional	Frail older people	Perceived (global)	MOS-SS	CES-D
Serrani 2014 [106] Argentina	100	48.2 (3.4) 51–55 *	91	Cross-sectional	Greater	Perceived (global)	Duke-UNC	CES-D
Serrano-Ortega 2017 [107] Spain	177	58.5 (12.9) 20–89	88	Longitudinal (repeated measures)	Frail older people	Perceived (global)	Duke-UNC	Goldberg
Shaughnessy 2011 [108] Canada	30	73.7 (6.9) 57–86	63.3	Cross-sectional	Dementia	Perceived (global)	MOS-SS	CES-D
Shukri 2020 [109] Malaysia	340	46 (15.2) 20–70	54.4	Cross-sectional	Hemodialysis	Perceived (global)	MSPSS	HADS
Speer 1993 [110] United States	26	67.3 (N/A) N/A	15	Cross-sectional	Parkinson	Perceived (emotional)	ISEL	GDS
Steffen 2002 [111] United States	145	60.2 (13.3) 33.6–86.8 *	80	Cross-sectional	Dementia	Perceived (global)	PSS	BDI
Stevens 2013 [112] Mexico	90	47.12 (12.7) 22–73 *	92	Cross-sectional	Traumatic brain injury	Perceived (global, emotional, instrumental)	ISEL	PHQ-9
Tay 2022 [12] United States	98	58.9 (14.24) 27–87	72.6	Cross-sectional	Palliative care	Perceived (global)	MOS-SS	HADS
Tang 2015 [113] United States	91	67 (12.2) 43–92 *	70	Cross-sectional	Dementia	Perceived (global)	ISEL	BDI-II
Thielemann 2001 [114] United States	164	61.9 (10.8) 31–81	60	Cross-sectional	Cancer	Perceived (global)	ISEL	CES-D
Verez Cotelo 2015 [115] Spain	25	55 (12.8) 39–87	80	Cross-sectional	Dementia	Perceived (global)	Duke-UNC	BDI-II
Yen 2006 [116] China	55	54.3 (14.7) 20–83	70.9	Cross-sectional	Mental health	Perceived (global)	Ad hoc	CES-D
Yoon 2003 [117] Korea	311	56.1 (15.6) 24–92	81	Cross-sectional	Frail older people	Received (global, emotional, instrumental)	PRQ-2000	SDS
Yun 2023 [118] Korea	396	80.7 (5.7) 69–96	57.6	Cross-sectional	Frail older people	Perceived (global)	Ad hoc	SGDS-K
Zhong 2020 [13] China	567	80.6 (8.8) 63–98 *	54.2	Cross-sectional	Frail older people	Perceived (global)	MSPSS	CES-D

Abbreviations: SD: standard deviation; *: ranges are estimated as ±2 standard deviations; N/A: not available; **: abbreviations of the scales used are shown in back matter part.

## 3. Results

### 3.1. Description of Search Results

**Figure 1:**
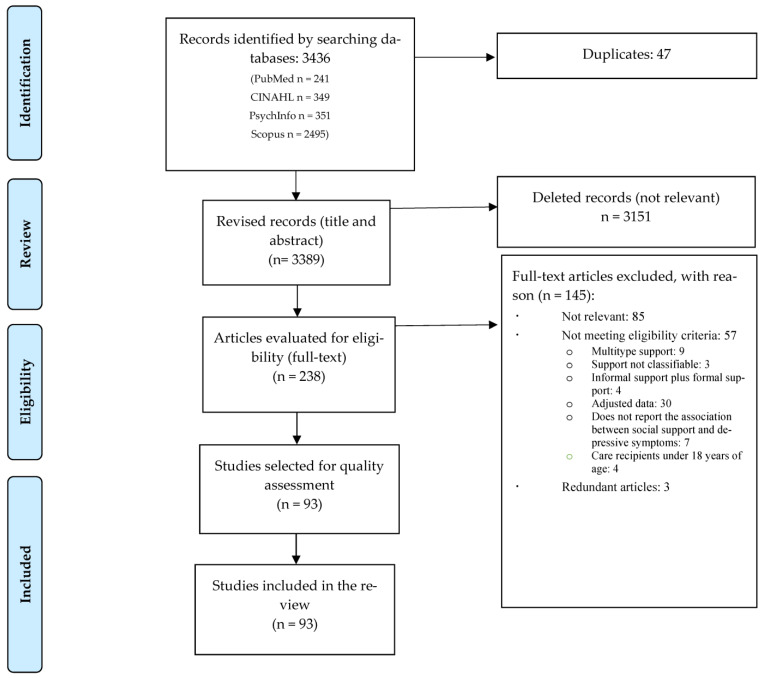
PRISMA flow diagram of the review process.

Our search yielded a total of 3436 results. After eliminating duplicates, 3389 results were reviewed by title and abstract. Of these, 3151 were eliminated for not being relevant, with a total of 238 full-texts being reviewed. After further screening, 85 articles were rejected as not relevant, with a total of 57 studies being excluded with specific reasons and 3 classified as redundant. Our final number of included studies was 93 [11,12,13,14,30,31,32,33,34,35,36,37,38,39,40,41,42,43,44,45,46,47,48,49,50,51,52,53,54,55,56,57,58,59,60,61,62,63,64,65,66,67,68,69,70,71,72,73,74,75,76,77,78,79,80,81,82,83,84,85,86,87,88,89,90,91,92,93,94,95,96,97,98,99,100,101,102,103,104,105,106,107,108,109,110,111,112,113,114,115,116,117,118] (Figure 1).

### 3.2. Description of Study Characteristics

As shown in Table 1, most of the studies included in the review, were cross-sectional, except for 6 studies which were longitudinal repeated measures (of which, two had cross-sectional correlations). Eighty-six percent of samples were non-probabilistic (*n* = 80). There was a total of 15,431 participants, with a minimum sample size of 17 and a maximum sample size of 691. The weighted average age of caregivers was 56.6 years, while the % of women was 74.6%. The year of publication ranged from 1987 to 2023. 

The most frequent chronic condition of care recipients was dementia (*n* = 26), followed by frailty (*n* = 21), cancer (*n* = 14), mental health condition (*n* = 5) and stroke (*n* = 5). Most studies measured levels of social support, with the Multidimensional Scale of Perceived Social Support (MSPSS) (perceived social support), and depressive symptoms using the Center for Epidemiological Studies (CES-D) scale.

### 3.3. Quality Assessment

Table 2 shows quality ratings of the included studies. All but thirteen studies used non-probability samples and twenty-one studies controlled for confounding. The majority of studies had no classification bias. All longitudinal studies, except one, reported attrition rates.

### 3.4. Results of the Different Meta-Analyses 

We were able to meta-analyse both the relationship of depressive symptoms with perceived social support and received social support. The results of the different meta-analyses are shown in Table 3.

#### 3.4.1. Perceived Social Support

**Figure 2:**
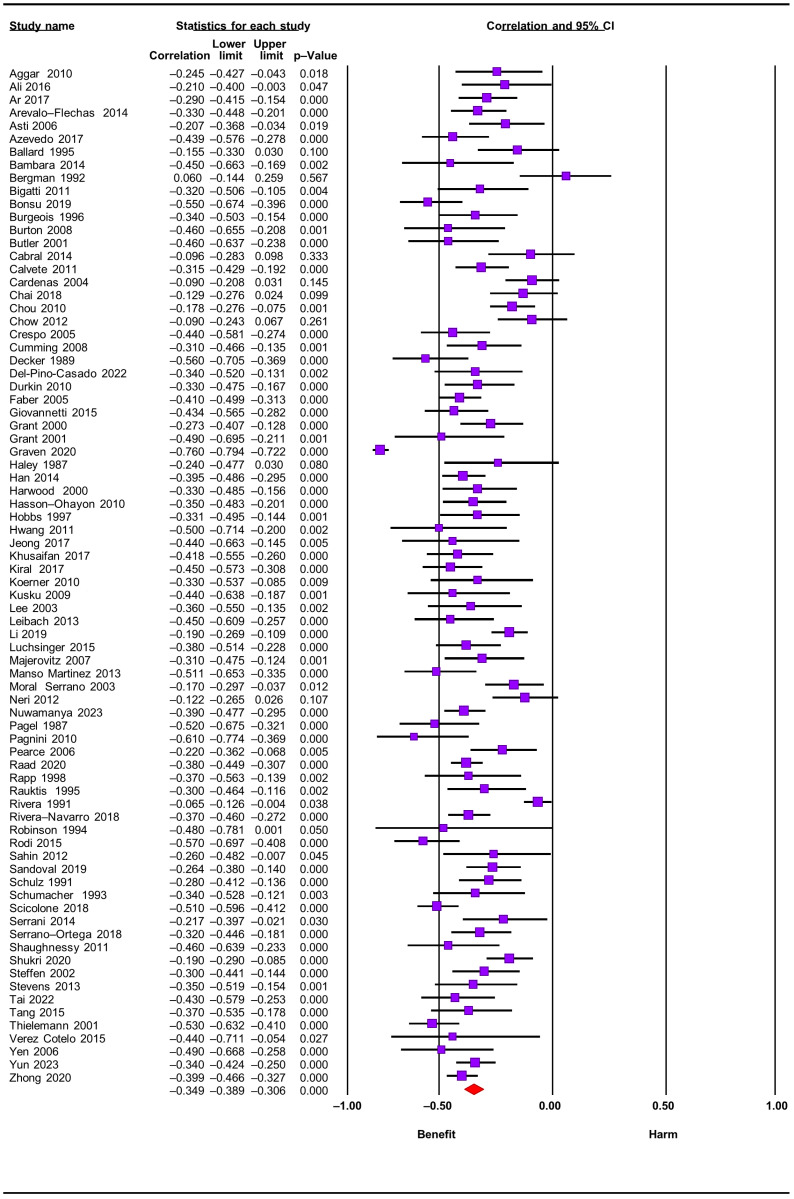
Forest plot for perceived social support and depressive symptoms [11,12,13,14,30,31,33,34,35,37,39,40,41,42,43,44,45,46,47,48,49,50,51,52,54,55,56,57,58,59,61,62,63,64,65,66,67,68,69,70,71,72,73,74,76,78,79,81,83,84,86,87,88,89,90,91,92,93,94,95,97,99,100,101,102,103,105,106,107,108,109,111,113,114,115,116,118].

**Figure 3:**
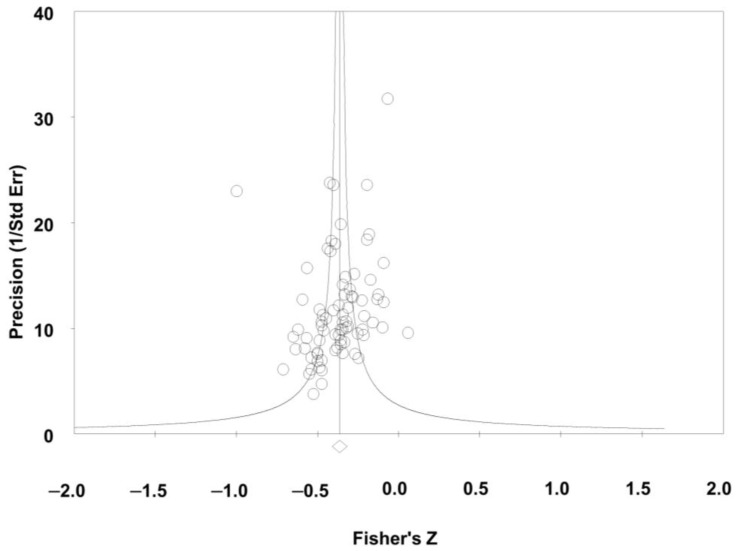
Funnel plot for perceived social support and depressive symptoms.

Seventy-eight studies reported on the relationship between global perceived social support and depressive symptoms; pooling studies showed a negative statistically significant association of a medium size effect (r = −0.35; confidence interval [CI] = −0.39; −0.31; N = 11.835; N average = 151.7; Figure 2). All but one study reported a negative direction and all but nine reported a negative statistical association. The results were consistent (I^2^ = 0.0; *p*-value for the Q test = 0.99), accurate, and robust (maximum percentage variation of the combined effect [% max] for sensitivity analysis eliminating one study at a time: = 5.7%). The funnel plot (Figure 3) appeared somewhat asymmetrical, with a small tendency for smaller studies to have larger effect sizes. The Egger test showed low risk of publication bias (*p* = 0.19) but the Trim & Fill test corrected the combined effect by 14.3% downwards.

After subgroup analyses, we found no statistically significant differences in effect sizes when taking into account type of study design (cross-sectional vs. longitudinal), type of chronic condition of the care recipient (frail older people, dementia, cancer, mental health disorder, and stroke) and study quality criteria (selection bias, classification, and confounding). Forest plots for subgroup analyses are shown in Figure S1. In addition, meta-regressions showed no variations of the combined effect due to caregiver mean age and % of females (*p*-values of 0.21 and 0.72, respectively). Scatterplots are shown in Supplementary Figures (Figure S2).

Regarding the dimensions of perceived social support, we found studies reporting on emotional and instrumental dimensions. Seven studies analysed the relationship between perceived emotional social support and depressive symptoms; meta-analysis showed a negative medium-size effect (r = −0.35; CI = −0.45; −0.25; N = 624; N mean = 89.1) with low heterogeneity overall (I^2^ = 19.1). The funnel plot appeared asymmetric, but the Trim and Fill method (corrected combined effect did not vary from the original) showed no publication bias.

Finally, three studies evaluated the relationship between instrumental perceived social support and depressive symptoms, with a negative and medium-sized association (r = −0.35; CI = −0.44; −0.26; N = 365; N average = 121.7), and no heterogeneity present (I^2^ = 0.0). We were unable to assess risk of publication bias due to the small number of included studies.

#### 3.4.2. Received Social Support

**Figure 4:**
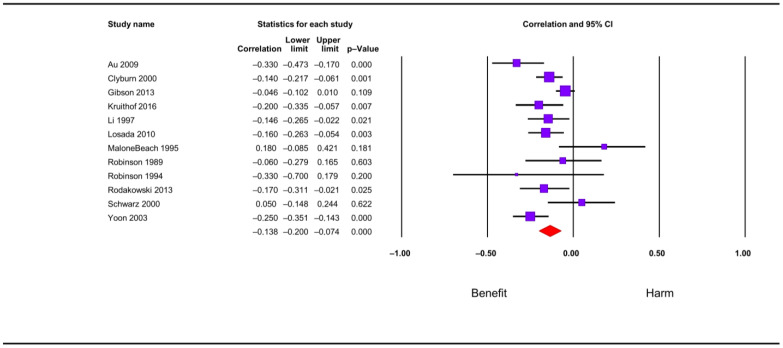
Forest plot for received social support and depressive symptoms [36,53,60,75,80,82,85,96,97,98,104,117].

**Figure 5:**
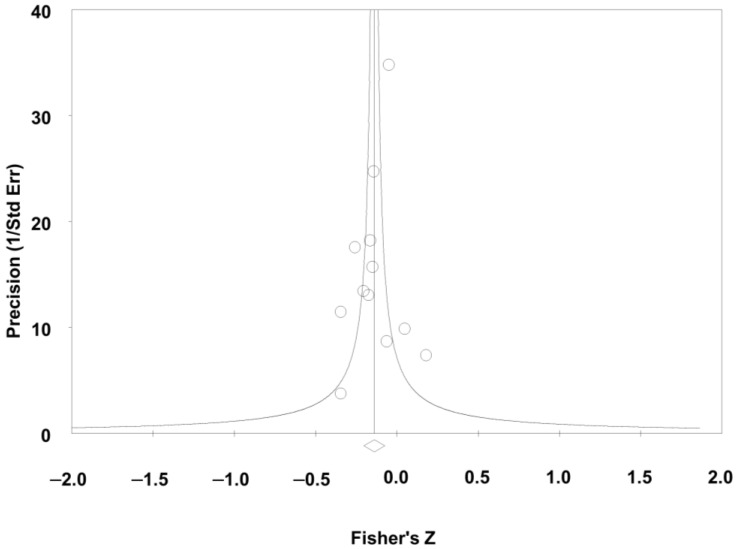
Funnel plot for received social support and depressive symptoms.

Twelve studies examined the relationship between global received social support and depressive symptoms; meta-analysis showed a negative statistical association of a small magnitude overall (r = −0.14; CI = −0.20; −0.07; N = 3470; N average = 289.2; Figure 4). All but two studies reported a negative direction and seven of the twelve studies reported a negative statistical association. Results showed overall low heterogeneity (I^2^ = 13.8; *p*-value for the Q test: 0.31) and were accurate and moderately robust (% max for sensitivity analysis eliminating one study at a time: 12.9%). Meta-regressions showed no variations of the combined effect due to caregiver mean age and % of females (*p*-value of 0.18 and 0.90, respectively). Scatterplots are shown in Supplementary Figures (Figure S2). The funnel plot (Figure 5) seemed somewhat asymmetric, although the Egger test showed no evidence of publication bias (*p* = 0.48), with the Trim & Fill test correcting the combined effect only by 7.1% downwards. 

Regarding the dimension of perceived social support, we found three studies reporting on emotionally received social support and three on instrumental support; meta-analysis indicated similar findings to global received social support (for emotional support: r = −0.15; CI = −0.23; −0.07; N = 620; N mean = 206.7; for instrumental: r = −0.14; CI = −0.26; −0.02; N = 620; N mean = 206.7).

## 4. Discussion

In the present work, we report on the first systematic review and meta-analysis of the relationship between social support and depressive symptoms of informal caregivers. We found that higher levels of depressive symptoms in informal caregivers of adult and older dependents are associated with lower levels of social support, both perceived and received dimensions. To our knowledge, our review is the first to systematically analyse this association, reporting on the global literature to date. An important strength of our review is that we report on a large number of studies overall, including data from diverse populations, with additional sensitivity and subgroup analyses allowing us to assess the effect of the quality of the evidence.

In relation to perceived social support, our results coincide with those of other reviews highlighting the possible protective effect of perceived social support on depressive symptoms in caregivers of children and adolescents with autism [119], in the general population [120], and healthy older people [121]. Our results are in line with reviews conducted in informal caregivers of dependent adults and older people reporting on the relationship between perceived social support and other mental health consequences of provision of care such as subjective caregiver burden [122] and anxiety symptoms [123]. Previous reviews [119,120,121], investigating the possible protective effect of perceived social support on depressive symptoms in populations other than those investigated in our review, also show similar findings.

The relationship between high levels of perceived social support and lower symptoms of depression could be explained under the prism of several different theories [9,10]. According to Lazarus and Folkman [9], in stressful situations, people carry out an assessment of both the possible repercussions of a situation and their ability to cope with the stressor. When these evaluations are negative, stress proliferates. In the caregiving context, if perceptions of social support are perceived as adequate, this may predispose carers to be more likely to cognitively assess the caregiving situation as benign and therefore perceive stressors as less threatening. This may in turn enhance confidence and self-efficacy in undertaking caregiving duties and positively influence mental health outcomes for carers.

Cohen and Wills [10,124] argued that social support may exert its effect at two different points in the causal sequence, linking stress to its consequences. On one hand, the perception that others can provide necessary support could lead to appraising a situation as less stressful. On the other hand, the support a person receives may lessen the influence of stress by facilitating problem-solving (providing a solution to the problem or reducing the perceived importance of the problem), providing a distraction from the distressing situation, or facilitating healthy behaviours. Therefore, when levels of social support are generally perceived as adequate, carer stress situations or associated stressors may appear as less threatening. This may therefore explain how high levels of social support can reduce levels of emotional distress such as depressive symptoms.

Our results reporting on the relationship between perceived social support and depressive symptoms are robust, based on sufficient levels of evidence overall. For example, we found adequate precision, and consistency, with no statistically significant differences between the combined effect of studies with good versus low quality. There were also no statistically significant differences between the different groups of recipients of care, and although there was evidence of risk of publication bias, the combined effect corrected by the Trim and Fill method varied little from the original combined effect. 

Our results therefore have important clinical implications as they indicate that levels of perceived social support by informal caregivers may be an important marker of depressive symptoms. Investing therefore in the development and provision of interventions promoting and strengthening perceived social support for families may prevent or alleviate carer depressive symptoms. For example, social support group interventions may improve carer well-being by reducing loneliness, and caregiver burden or enhance the perceived effectiveness of how carers manage caregiving tasks [125].

Our results on the relationship between received social support and depressive symptoms are moderately robust with sufficient levels of evidence overall since there was adequate precision, low inconsistency and, although publication bias was present, this risk had little effect on the final results. Due to the small number of included studies, however, we were not able to perform subgroup analyses.

Interestingly as in previous reviews [122,123], we found that the effect of received social support on depressive symptoms was overall small, suggesting that the central role of social support on the onset and development of depressive symptoms in informal caregivers may be specific to perceived rather than received levels of social support, similar to previous reviews [122,123].

Our results therefore support the hypothesis that perceived and received levels of social support are different constructs with different effects on stress proliferation [126]. For example, several studies have shown that the effect of received support on stress proliferation is smaller compared to that of perceived social support [127,128]. Our study expands knowledge on caregiver depressive symptoms by demonstrating how different types of social support contribute to the experience of depressive affect. The findings from this work can therefore inform interventions that target specific areas of support for family carers and those that aim to reduce the risk of mild symptoms of depression developing into clinical psychopathology. Our results may also provide valuable insights for policymakers, clinicians, and researchers as they point towards the value of implementing social support interventions as preventive mental health strategies for carers. Future studies should assess how different dimensions of social support impact caregivers’ mental health and investigate how cultural or contextual factors might influence the relationship between social support and depressive symptoms. Future work should also examine how other social parameters such as social recognition may be influencing the association between social support and carer depressive symptoms.

### Limitations

Our study has several limitations. Most studies included in our review employed a cross-sectional design and used non-probability samples. Cross-sectional designs are limited in informing causality relationships. However, given our subgroup analyses showed that this did not affect our results we are relatively confident that our reported effect estimates are close to the true effect estimate. In the case of received social support, subgroup analyses could not be carried out due to the small number of studies; this means that the effect of type of design, study quality, or cause of care-recipient dependency on the relationship between received social support and depressive symptoms remains unknown. It was also not possible to control for several confounders such as a prior history of depression or analyse the effect of objective caregiver burden for received social support. Finally, the longitudinal studies included in this review did not report analyses controlling for possible reverse causality. The existence of reverse causality would imply that depressive symptoms could influence perceptions of social support. Future longitudinal studies therefore are necessary to examine the effect of reverse causality, which will increase our confidence in the estimated effect sizes. 

## 5. Conclusions

Despite the above limitations, we are able to reach important clinical conclusions about levels of social support in informal caregivers of dependent adults and older people: (1) perceived social support and received social support are different constructs that differentially influence depressive symptoms in informal caregivers, (2) perceived social support is an important protective factor for high levels of depressive symptoms reported by informal carers, (3) the relationship between perceived social support and depressive symptoms does not vary substantially across the different types of care dependency and (4) the effect of perceived social support on depressive symptoms appears to be clinically relevant.

Our results overall strengthen the use of social support as a marker of clinically significant depressive symptoms for informal carers. They also support the development and wider provision of interventions promoting and strengthening perceived social support to prevent or alleviate depressive symptoms in informal caregivers. 

Further longitudinal studies analysing the possible effect of reverse causality between social support and depressive symptoms are needed to increase our understanding of the effect of social support on caregivers’ mental health.

## Figures and Tables

**Table 2:** Quality assessment of included studies.

Author and Year	Subgroup	Type	Selection	Classification	Confounding	Attrition
Aggar 2010 [30]			−	+	?	NA
Ali 2016 [31]			−	+	+	NA
Amorim 2009 [32]			−	+	?	NA
Ar 2017 [32]			−	+	?	NA
Arevalo-Flechas 2014 [34]			−	+	?	NA
Asti 2006 [35]			−	+	?	NA
Au 2009 [36]			−	+	+	NA
Azevedo 2017 [37]			−	+	+	NA
Baillie 1988 [38]			−	+	?	NA
Ballard 1995 [39]			−	+	?	NA
Bambara 2014 [40]			−	+	+	NA
Bergman 1992 [41]			−	+	?	NA
Bigatti 2011 [42]			−	+	?	NA
Bonsu 2019 [43]			−	+	?	NA
Burgeois 1996 [44]			−	+	?	NA
Burton 2008 [45]			−	+	+	NA
Butler 2001 [46]			−	+	+	NA
Cabral 2014 [47]			−	+	+	NA
Calvete 2011 [48]			−	+	?	NA
Cardenas 2004 [49]			−	+	?	NA
Chai 2018 [50]			−	+	?	NA
Chou 2010 [51]			−	+	?	NA
Chow 2012 [52]			−	+	−	NA
Clyburn 2000 [53]			+	+	?	NA
Crespo 2005 [54]			−	+	?	NA
Cumming 2008 [55]			−	+	?	NA
Decker 1989 [56]			−	?	?	NA
Del-Pino-Casado 2022 [57]			+	+	+	+
Durkin 2010 [58]			−	+	?	?
Faber 2005 [59]			−	+	?	NA
Gibson 2013 [60]			−	+	−	NA
Giovannetti 2015 [61]			−	+	?	NA
Grant 2000 [62]			−	+	?	NA
Grant 2001 [63]			−	+	?	NA
Graven 2020 [64]			−	+	+	NA
Haley 1987 [65]			−	+	?	NA
Han 2014 [66]			−	+	?	NA
Harwood 2000 [67]			−	+	+	NA
Hasson-Ohayon 2010 [68]			−	+	?	NA
Hobbs 1997 [69]			+	+	?	NA
Hwang 2011 [70]			−	+	?	NA
Jeong 2017 [71]			−	+	?	NA
Khusaifan 2017 [72]			−	+	?	NA
Kiral 2017 [73]			−	+	?	NA
Koerner 2010 [74]			−	+	+	NA
Kruithof 2016 [75]			−	+	?	+
Kusku 2009 [76]			−	+	?	NA
Lakey 2002 [77]			−	+	?	NA
Lee 2003 [78]			+	+	+	NA
Leibach 2013 [79]			−	+	−	NA
Li 1997 [80]	Daughter	Emotional	+	+	+	NA
Li 1997 [80]	Daughter	Global	+	+	+	NA
Li 1997 [80]	Daughter	Instrumental	+	+	−	NA
Li 1997 [80]	Wife	Emotional	+	+	+	NA
Li 1997 [80]	Wife	Global	+	+	+	NA
Li 1997 [80]	Wife	Instrumental	+	+	−	NA
Li 2019 [81]			+	+	+	NA
Losada 2010 [82]			−	+	?	NA
Luchsinger 2015 [83]			−	+	?	NA
Majerovitz 2007 [84]			−	+	?	NA
MaloneBeach 1995 [85]			−	+	?	NA
Manso Martinez 2013 [86]	Men		−	+	−	NA
Manso Martinez 2013 [86]	Women		−	+	+	NA
Moral Serrano 2003 [87]			+	+	?	NA
Neri 2012 [88]			−	+	?	NA
Nuwamanya 2023 [11]			−	+	?	NA
Pagel 1987 [89]			−	+	?	NA
Pagnini 2010 [90]			−	+	+	NA
Pearce 2006 [91]			−	+	?	NA
Raad 2020 [14]			−	+	?	NA
Rapp 1998 [92]			−	+	−	NA
Rauktis 1995 [93]			+	+	−	NA
Rivera 1991 [94]			−	+	?	NA
Rivera-Navarro 2018 [95]			−	+	+	NA
Robinson 1989 [96]			−	+	?	NA
Robinson 1994 [97]			−	+	?	NA
Rodakowski 2013 [98]			+	+	?	NA
Rodi 2015 [99]			−	+	?	NA
Sahin 2012 [100]			−	+	?	NA
Sandoval 2019 [101]			−	+	−	NA
Schulz 1991 [102]			−	+	?	NA
Schumacher 1993 [103]			−	+	−	NA
Schwarz 2000 [104]			−	+	?	NA
Scicolone 2018 [105]			−	+	+	NA
Serrani 2014 [106]			+	+	?	NA
Serrano-Ortega 2017 [107]			+	+	+	+
Shaughnessy 2011 [108]			−	+	?	NA
Shukri 2020 [109]			−	+	?	NA
Speer 1993 [110]			−	+	?	NA
Steffen 2002 [111]			−	+	?	NA
Stevens 2013 [112]			−	+	+	NA
Tay 2022 [12]			−	+	?	NA
Tang 2015 [113]			−	+	+	NA
Thielemann 2001 [114]			−	+	+	NA
Verez Cotelo 2015 [115]			−	+	?	NA
Yen 2006 [116]			−	+	?	NA
Yoon 2003 [117]		Emotional	−	+	−	NA
Yoon 2003 [117]		Global	−	+	?	NA
Yoon 2003 [117]		Instrumental	−	+	−	NA
Yun 2023 [118]			+	?	+	NA
Zhong 2020 [13]			+	+	?	NA

Abbreviations: NA: Not applicable; (−) Risk of bias; (+) Low risk of bias; (?) Not enough information to evaluate.

**Table 3:** Findings of the meta-analyses on the relationship between social support and depressive symptoms.

Type of Social Support	Global/ Dimensions	Whole Sample/Subgroups		K	N	Average N	Combined Effect			Heterogeneity			Sensitivity Analyses		Publication Bias			
							r	Lower Limit	Upper Limit	Q (df)	*p*	I^2^			Funnel	Egger’s	Trim & Fill	
		Criterion	Categories										r Max	%		*p*-Value	r	%
Perceived	Global	Whole sample	--	78	11,835	151.7	−0.35	−0.39	−0.31	49.6 (77)	0.99	0.0	−0.33	5.7	Asym	0.19	−0.3	14.3
		Type of care-recipient	Dementia	20	2507	125.4	−0.31	−0.37	−0.26	18.4 (19)	0.5	0.0	−0.33	5.2	Asym	0.38	−0.29	7.6
			Frail older p	17	1753	103.1	−0.30	−0.38	−0.23	12.1 (16)	0.73	0.0	−0.29	4.7	Asym	0.08	−0.3	0.0
			Cancer	13	1813	139.5	−0.37	−0.44	−0.31	11.1 (12)	0.52	0.0	−0.39	5.4	Sym	0.53	−0.37	0.0
			Mental Illness	5	530	106.0	−0.26	−0.38	−0.12	4.4 (4)	0.35	9.4	−0.21	19.2	NV	NV	NV	NV
			Stroke	4	298	74.5	−0.29	−0.38	−0.20	2.8 (3)	9.42	0.0	−0.27	7.4	NV	NV	NV	NV
		Design	Cross-sectional	75	11,447	152.6	−0.35	−0.39	−0.31	48.0 (74)	0.99	0.0	−0.33	4.9	Asym	0.2	−0.3	14.3
			Longitudinal	3	388	129.3	−0.33	−0.41	−0.24	0.029 (2)	0.99	0.0	−0.32	1.1	NV	NV	NV	NV
		Sampling	Prob	10	2368	236.8	−0.30	−0.36	−0.23	6.2 (9)	0.72	0.0	−0.32	4.8	Asym	0.95	−0.27	11.3
			Non-prob	68	9467	139.2	−0.36	−0.40	−0.31	40.7 (67)	0.99	0.0	−0.34	4.1	Asym	0.22	−0.3	15.7
		Control of confounders	Yes	20	876	43.8	−0.40	−0.50	−0.29	9.3 (19)	0.97	0.0	−0.37	8.9	Asym	0.45	−0.45	11.7
			No	58	10,959	188.9	−0.32	−0.36	−0.29	49.3 (57)	0.76	0.0	−0.33	1.5	Asym	0.002	−0.29	10.2
	Emotional	Whole sample	--	7	624	89.1	−0.35	−0.45	−0.25	7.4 (6)	0.29	19.1	−0.38	9.4	Asym	NV	−0.35	0.0
	Instrumental	Whole sample	--	3	365	121.7	−0.35	−0.44	−0.26	0.012 (2)	0.99	0.0	−0.36	0.8	NV	NV	NV	NV
Received	Global	Whole sample	--	12	3470	289.2	−0.14	−0.20	−0.07	12.8 (11)	0.31	13.8	−0.12	12.9	Asym	0.48	−0.13	7.1
	Emotional	Whole sample	--	3	620	206.7	−0.15	−0.23	−0.07	1.4 (2)	0.5	0.0	−0.20	28.2	NV	NV	NV	NV
	Instrumental	Whole sample	--	3	620	206.7	−0.14	−0.26	−0.02	2.0 (2)	0.37	1.9	−0.20	36.3	NV	NV	NV	NV

Abbreviations: K: number of studies; N: sample size; r: combined correlation coefficient; r max: maximum value of the combined effect for sensitivity analysis eliminating one study at a time; %: percentage of variation from the original combined effect; Prob: probability sampling; Non-prob: Non-probability sampling; Frail older p: frail older people; Asym: asymmetric; Sym: symmetrical; NV: not valuable (its assessment is not recommended when there are few studies).

## Data Availability

The data presented in this study are available on request from the corresponding author.

## Supplementary Materials

The following supporting information can be downloaded at: https://www.mdpi.com/article/10.3390/jcm12206468/s1, Table S1: Summary of the search strategy used in the databases. Figure S1: Forest plots for subgroup analyses. Figure S2: Scatterplots for meta-regressions.

## Abbreviations

Abbreviations of Measures of:	
Social Support	
ASSIS	Arizona Social Support Interview Schedule
CRA	Caregiver Reaction Assessment
CPASS	Cancer Perceived Agent of Support Questionnaire
Duke-UNC	Duke-UNC functional social support questionnaire
ESSS	Satisfaction with Social Support Scale
FNQ	Family Needs Questionnaire
GSS	Global Satisfaction Scale
HDLS	Health and Daily Living Form
ISEL	Interpersonal Support Evaluation List
ISSB	Inventory of Socially Supportive Behavior
K & B-C	Krause and Borawski-Clark Scale
MG-SS	McGill Quality of Life- Social Support
MISSB	Modified Inventory of Social Support Behaviors
MOS-SS	Medical Outcome Study- Social Support Survey
MSPSS	Multidimensional Scale of Perceived Social Support
MSSS	Mac-Arthur Social Support Scale
NSIS	Negative Social Interaction
PESS	Pearling Expressive Support Scale
PRQ-85	Personal Resource Questionnaire
PSI	Positive Social Interactions
PSS	Perceived Social Support Scale
PSQ	Psychosocial Support Questionnaire
QRI	Quality of Relationships Inventory
SOCSUP	Total Social Support Scale Score
SNL	Social Network List
SSL	Social Support List
SSNL	Stokes’ Social Network List
SSSQ	Sarason’s Social Support Questionnaire
SSRS	Social Support Rating Scale
SSS	Social Support Scale
SSSI	Social Stress and Social Interview
TBI-CareQOL	The Traumatic Injury Quality of Life
Walen & Lanchman	Walen & Lanchman Questionnaire
Depression	
BDI	Beck Depression Inventory
BSI	Brief Symptom Inventory
CES-D	The Center for Epidemiological Studies—Depression Scale
Cornell	Cornell Depression scale
DASS-42	Depression Anxiety and Stress Scale
DSM-IV	Mayor Depressive Disorder and Anxiety Disorder Diagnoses by the Structured Clinical Interview.
EADS-21	Escalas de Ansiedad, Depresión y Estrés [Anxiety, Depression and Stress Scales]
GDS	Global Deterioration Scale
GHQ	General Health Questionnaire
HADS	Hospital Anxiety and Depression Scale
HDRS	Hamilton Depression Rating Scale
HSC	Hopkins Symptom Checklist
IDA Scale	Irritability, Depression, and Anxiety Scale
PHQ-9	Patient Health Questionnaire
POMS	Profile of Mood States
QIDS-SR 16	Quick Inventory of Depressive Symptomatology
SCL-90	Symptom Checklist
SDS	Self-Rating Depression Scale
SQD	Square Depression Scale
TBI-CareQOL	The Traumatic Injury Quality of Life
