# Supplementary material for: Association between Social Support and Depressive Symptoms in Informal Caregivers of Adult and Older Dependents: A Systematic Review and Meta-Analysis
Source: J Clin Med. 2023 Oct 11;12(20):6468. doi: 10.3390/jcm12206468 (PMC10607501; doi:10.3390/jcm12206468)

FOREST PLOTS FOR SUBGROUP ANALYSES

Forest plot for subgroups by design

Group by Design Dic	Study name	Subgroup within study	Comparison	Outcome	Statistics for each study: Correlation	Lower limit	Upper limit	p-Value
CS	Aggar 2010	Blank	Perceived	Global	-0,245	-0,427	-0,043	0,018
CS	Ali 2016	Blank	Perceived	Global	-0,210	-0,400	-0,003	0,047
CS	Ar 2017	Blank	Perceived	Global	-0,290	-0,415	-0,154	0,000
CS	Arevalo-Flechas 2014	Blank	Perceived	Global	-0,330	-0,448	-0,201	0,000
CS	Asti 2006	Combined	Perceived	Global	-0,207	-0,368	-0,034	0,019
CS	Azevedo 2017	Blank	Perceived	Global	-0,439	-0,576	-0,278	0,000
CS	Ballard 1995	Blank	Perceived	Global	-0,155	-0,330	0,030	0,100
CS	Bambara 2014	Blank	Perceived	Global	-0,450	-0,663	-0,169	0,002
CS	Bergman 1992	Blank	Perceived	Global	0,060	-0,144	0,259	0,567
CS	Bigatti 2011	Blank	Perceived	Global	-0,320	-0,506	-0,105	0,004
CS	Bonsu 2019	Blank	Perceived	Global	-0,550	-0,674	-0,396	0,000
CS	Burgeois 1996	Blank	Perceived	Global	-0,340	-0,503	-0,154	0,000
CS	Burton 2008	Blank	Perceived	Global	-0,460	-0,655	-0,208	0,001
CS	Butler 2001	Blank	Perceived	Global	-0,460	-0,637	-0,238	0,000
CS	Cabral 2014	Blank	Perceived	Global	-0,096	-0,283	0,098	0,333
CS	Calvete 2011	Blank	Perceived	Global	-0,315	-0,429	-0,192	0,000
CS	Cardenas 2014	Blank	Perceived	Global	-0,090	-0,208	0,031	0,145
CS	Chai 2018	Blank	Perceived	Global	-0,129	-0,276	0,024	0,099
CS	Chou 2010	Blank	Perceived	Global	-0,178	-0,276	-0,075	0,001
CS	Chow 2012	Blank	Perceived	Global	-0,090	-0,243	0,067	0,261
CS	Crespo 2005	Blank	Perceived	Global	-0,440	-0,581	-0,274	0,000
CS	Cumming 2008	Blank	Perceived	Global	-0,310	-0,466	-0,135	0,001
CS	Decker 1989	Blank	Perceived	Global	-0,560	-0,705	-0,369	0,000
CS	Faber 2005	Blank	Perceived	Global	-0,410	-0,499	-0,313	0,000
CS	Giovannetti 2015	Blank	Perceived	Global	-0,434	-0,565	-0,282	0,000
CS	Grant 2000	Blank	Perceived	Global	-0,273	-0,407	-0,128	0,000
CS	Grant 2001	Blank	Perceived	Global	-0,490	-0,695	-0,211	0,001
CS	Graven 2020	Blank	Perceived	Global	-0,760	-0,794	-0,722	0,000
CS	Haley 1987	Blank	Perceived	Global	-0,240	-0,477	0,030	0,080
CS	Han 2014	Blank	Perceived	Global	-0,395	-0,486	-0,295	0,000
CS	Harwood 2000	Blank	Perceived	Global	-0,330	-0,485	-0,156	0,000
CS	Hasson-Ohayon 2010	Blank	Perceived	Global	-0,350	-0,483	-0,201	0,000
CS	Hobbs 1997	Blank	Perceived	Global	-0,331	-0,495	-0,144	0,001
CS	Hwang 2011	Blank	Perceived	Global	-0,500	-0,714	-0,200	0,002
CS	Jeong 2017	Blank	Perceived	Global	-0,440	-0,663	-0,145	0,005
CS	Khusaifan 2017	Blank	Perceived	Global	-0,418	-0,555	-0,260	0,000
CS	Kiral 2017	Blank	Perceived	Global	-0,450	-0,573	-0,308	0,000
CS	Koerner 2010	Blank	Perceived	Global	-0,330	-0,537	-0,085	0,009
CS	Kusku 2009	Blank	Perceived	Global	-0,440	-0,638	-0,187	0,001
CS	Lee 2003	Blank	Perceived	Global	-0,360	-0,550	-0,135	0,002
CS	Leibach 2013	Blank	Perceived	Global	-0,450	-0,609	-0,257	0,000
CS	Li 2019	Blank	Perceived	Global	-0,190	-0,269	-0,109	0,000
CS	Luchsinger 2015	Blank	Perceived	Global	-0,380	-0,514	-0,228	0,000
CS	Majerovitz 2007	Blank	Perceived	Global	-0,310	-0,475	-0,124	0,001
CS	Manso Martinez 2013	Combined	Perceived	Global	-0,511	-0,653	-0,335	0,000
CS	Moral Serrano 2003	Blank	Perceived	Global	-0,170	-0,297	-0,037	0,012
CS	Neri 2012	Blank	Perceived	Global	-0,122	-0,265	0,026	0,107
CS	Nuwamanya 2023	Blank	Perceived	Global	-0,390	-0,477	-0,295	0,000
CS	Pagel 1987	Blank	Perceived	Global	-0,520	-0,675	-0,321	0,000
CS	Pagnini 2010	Blank	Perceived	Global	-0,610	-0,774	-0,369	0,000
CS	Pearce 2006	Blank	Perceived	Global	-0,220	-0,362	-0,068	0,005
CS	Raad 2020	Blank	Perceived	Global	-0,380	-0,449	-0,307	0,000
CS	Rapp 1998	Blank	Perceived	Global	-0,370	-0,563	-0,139	0,002
CS	Rauktis 1995	Blank	Perceived	Global	-0,300	-0,464	-0,116	0,002
CS	Rivera 1991	Blank	Perceived	Global	-0,065	-0,126	-0,004	0,038
CS	Rivera-Navarro 2018	Blank	Perceived	Global	-0,370	-0,460	-0,272	0,000
CS	Robinson 1994	Blank	Perceived	Global	-0,480	-0,781	0,001	0,050
CS	Rodi 2015	Blank	Perceived	Global	-0,570	-0,697	-0,408	0,000
CS	Sahin 2012	Blank	Perceived	Global	-0,260	-0,482	-0,007	0,045
CS	Sandoval 2019	Blank	Perceived	Global	-0,264	-0,380	-0,140	0,000
CS	Schulz 1991	Blank	Perceived	Global	-0,280	-0,412	-0,136	0,000
CS	Schumacher 1993	Blank	Perceived	Global	-0,340	-0,528	-0,121	0,003
CS	Scicolone 2018	Blank	Perceived	Global	-0,510	-0,596	-0,412	0,000
CS	Serrani 2014	Blank	Perceived	Global	-0,217	-0,397	-0,021	0,030
CS	Shaughnessy 2011	Blank	Perceived	Global	-0,460	-0,639	-0,233	0,000
CS	Shukri 2020	Blank	Perceived	Global	-0,190	-0,290	-0,085	0,000
CS	Steffen 2002	Blank	Perceived	Global	-0,300	-0,441	-0,144	0,000
CS	Stevens 2013	Blank	Perceived	Global	-0,350	-0,519	-0,154	0,001
CS	Tai 2021	Blank	Perceived	Global	-0,430	-0,579	-0,253	0,000
CS	Tang 2015	Blank	Perceived	Global	-0,370	-0,535	-0,178	0,000
CS	Thielemann 2001	Blank	Perceived	Global	-0,530	-0,632	-0,410	0,000
CS	Verez Cotelo 2015	Blank	Perceived	Global	-0,440	-0,711	-0,054	0,027
CS	Yen 2006	Blank	Perceived	Global	-0,490	-0,668	-0,258	0,000
CS	Yun 2023	Blank	Perceived	Global	-0,340	-0,424	-0,250	0,000
CS	Zhong 2020	Blank	Perceived	Global	-0,399	-0,466	-0,327	0,000
CS					-0,350	-0,392	-0,306	0,000
RML	Del-Pino-Casado 2022	Blank	Perceived	Global	-0,340	-0,520	-0,131	0,002
RML	Durkin 2010	Blank	Perceived	Global	-0,330	-0,475	-0,167	0,000
RML	Serrano-Ortega 2018	Blank	Perceived	Global	-0,320	-0,446	-0,181	0,000
RML					-0,327	-0,414	-0,235	0,000

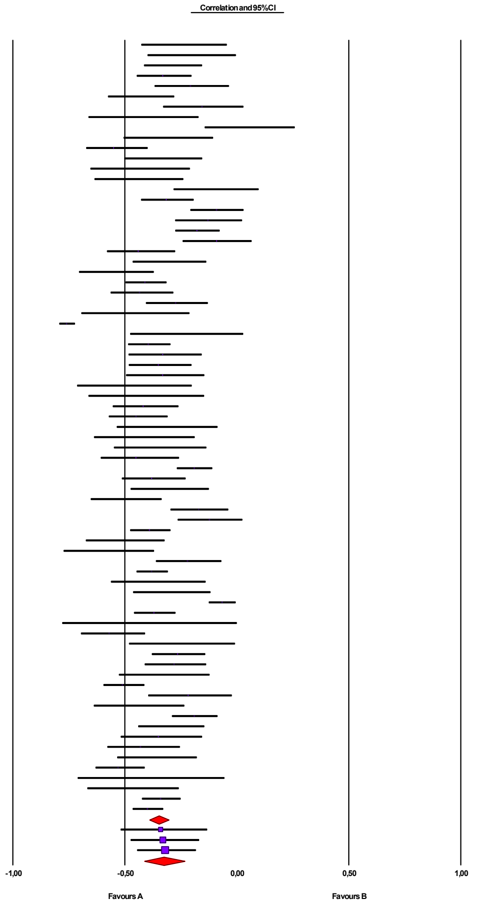

Abbreviations: CS: cross-sectional design or repeated measures design with cross-sectional correlations; RML: longitudinal (repeated measures design with longitudinal correlations).

Forest plot for subgroups by sampling

Group by Sampling	Study name	Subgroup within study	Comparison	Outcome	Correlation	Lower limit	Upper limit	p-Value
Non-probabilistic	Aggar 2010	Blank	Perceived	Global	-0,245	-0,427	-0,043	0,018
Non-probabilistic	Ali 2016	Blank	Perceived	Global	-0,210	-0,400	-0,003	0,047
Non-probabilistic	Ar 2017	Blank	Perceived	Global	-0,290	-0,415	-0,154	0,000
Non-probabilistic	Arevalo-Flechas 2014	Blank	Perceived	Global	-0,330	-0,448	-0,201	0,000
Non-probabilistic	Asti 2006	Combined	Perceived	Global	-0,207	-0,368	-0,034	0,019
Non-probabilistic	Azevedo 2017	Blank	Perceived	Global	-0,439	-0,576	-0,278	0,000
Non-probabilistic	Ballard 1995	Blank	Perceived	Global	-0,155	-0,330	0,030	0,100
Non-probabilistic	Bambara 2014	Blank	Perceived	Global	-0,450	-0,663	-0,169	0,002
Non-probabilistic	Bergman 1992	Blank	Perceived	Global	0,060	-0,144	0,259	0,567
Non-probabilistic	Bigatti 2011	Blank	Perceived	Global	-0,320	-0,506	-0,105	0,004
Non-probabilistic	Bonsu 2019	Blank	Perceived	Global	-0,550	-0,674	-0,396	0,000
Non-probabilistic	Burgeois 1996	Blank	Perceived	Global	-0,340	-0,503	-0,154	0,000
Non-probabilistic	Burton 2008	Blank	Perceived	Global	-0,460	-0,655	-0,208	0,001
Non-probabilistic	Butler 2001	Blank	Perceived	Global	-0,460	-0,637	-0,238	0,000
Non-probabilistic	Cabral 2014	Blank	Perceived	Global	-0,096	-0,283	0,098	0,333
Non-probabilistic	Calvete 2011	Blank	Perceived	Global	-0,315	-0,429	-0,192	0,000
Non-probabilistic	Cardenas 2014	Blank	Perceived	Global	-0,090	-0,208	0,031	0,145
Non-probabilistic	Chai 2018	Blank	Perceived	Global	-0,129	-0,276	0,024	0,099
Non-probabilistic	Chou 2010	Blank	Perceived	Global	-0,178	-0,276	-0,075	0,001
Non-probabilistic	Chow 2012	Blank	Perceived	Global	-0,090	-0,243	0,067	0,261
Non-probabilistic	Crespo 2005	Blank	Perceived	Global	-0,440	-0,581	-0,274	0,000
Non-probabilistic	Cumming 2008	Blank	Perceived	Global	-0,310	-0,466	-0,135	0,001
Non-probabilistic	Decker 1989	Blank	Perceived	Global	-0,560	-0,705	-0,369	0,000
Non-probabilistic	Durkin 2010	Blank	Perceived	Global	-0,330	-0,475	-0,167	0,000
Non-probabilistic	Faber 2005	Blank	Perceived	Global	-0,410	-0,499	-0,313	0,000
Non-probabilistic	Giovannetti 2015	Blank	Perceived	Global	-0,434	-0,565	-0,282	0,000
Non-probabilistic	Grant 2000	Blank	Perceived	Global	-0,273	-0,407	-0,128	0,000
Non-probabilistic	Grant 2001	Blank	Perceived	Global	-0,490	-0,695	-0,211	0,001
Non-probabilistic	Graven 2020	Blank	Perceived	Global	-0,760	-0,794	-0,722	0,000
Non-probabilistic	Haley 1987	Blank	Perceived	Global	-0,240	-0,477	0,030	0,080
Non-probabilistic	Han 2014	Blank	Perceived	Global	-0,395	-0,486	-0,295	0,000
Non-probabilistic	Harwood 2000	Blank	Perceived	Global	-0,330	-0,485	-0,156	0,000
Non-probabilistic	Hasson-Ohayon 2010	Blank	Perceived	Global	-0,350	-0,483	-0,201	0,000
Non-probabilistic	Hwang 2011	Blank	Perceived	Global	-0,500	-0,714	-0,200	0,002
Non-probabilistic	Jeong 2017	Blank	Perceived	Global	-0,440	-0,663	-0,145	0,005
Non-probabilistic	Khusaifan 2017	Blank	Perceived	Global	-0,418	-0,555	-0,260	0,000
Non-probabilistic	Kiral 2017	Blank	Perceived	Global	-0,450	-0,573	-0,308	0,000
Non-probabilistic	Koerner 2010	Blank	Perceived	Global	-0,330	-0,537	-0,085	0,009
Non-probabilistic	Kusku 2009	Blank	Perceived	Global	-0,440	-0,638	-0,187	0,001
Non-probabilistic	Leibach 2013	Blank	Perceived	Global	-0,450	-0,609	-0,257	0,000
Non-probabilistic	Luchsinger 2015	Blank	Perceived	Global	-0,380	-0,514	-0,228	0,000
Non-probabilistic	Majerovitz 2007	Blank	Perceived	Global	-0,310	-0,475	-0,124	0,001
Non-probabilistic	Manso Martinez 2013	Combined	Perceived	Global	-0,511	-0,653	-0,335	0,000
Non-probabilistic	Neri 2012	Blank	Perceived	Global	-0,122	-0,265	0,026	0,107
Non-probabilistic	Nuwamanya 2023	Blank	Perceived	Global	-0,390	-0,477	-0,295	0,000
Non-probabilistic	Pagel 1987	Blank	Perceived	Global	-0,520	-0,675	-0,321	0,000
Non-probabilistic	Pagnini 2010	Blank	Perceived	Global	-0,610	-0,774	-0,369	0,000
Non-probabilistic	Pearce 2006	Blank	Perceived	Global	-0,220	-0,362	-0,068	0,005
Non-probabilistic	Raad 2020	Blank	Perceived	Global	-0,380	-0,449	-0,307	0,000
Non-probabilistic	Rapp 1998	Blank	Perceived	Global	-0,370	-0,563	-0,139	0,002
Non-probabilistic	Rivera 1991	Blank	Perceived	Global	-0,065	-0,126	-0,004	0,038
Non-probabilistic	Rivera-Navarro 2018	Blank	Perceived	Global	-0,370	-0,460	-0,272	0,000
Non-probabilistic	Robinson 1994	Blank	Perceived	Global	-0,480	-0,781	0,001	0,050
Non-probabilistic	Rodi 2015	Blank	Perceived	Global	-0,570	-0,697	-0,408	0,000
Non-probabilistic	Sahin 2012	Blank	Perceived	Global	-0,260	-0,482	-0,007	0,045
Non-probabilistic	Sandoval 2019	Blank	Perceived	Global	-0,264	-0,380	-0,140	0,000
Non-probabilistic	Schulz 1991	Blank	Perceived	Global	-0,280	-0,412	-0,136	0,000
Non-probabilistic	Schumacher 1993	Blank	Perceived	Global	-0,340	-0,528	-0,121	0,003
Non-probabilistic	Scicolone 2018	Blank	Perceived	Global	-0,510	-0,596	-0,412	0,000
Non-probabilistic	Shaughnessy 2011	Blank	Perceived	Global	-0,460	-0,639	-0,233	0,000
Non-probabilistic	Shukri 2020	Blank	Perceived	Global	-0,190	-0,290	-0,085	0,000
Non-probabilistic	Steffen 2002	Blank	Perceived	Global	-0,300	-0,441	-0,144	0,000
Non-probabilistic	Stevens 2013	Blank	Perceived	Global	-0,350	-0,519	-0,154	0,001
Non-probabilistic	Tai 2021	Blank	Perceived	Global	-0,430	-0,579	-0,253	0,000
Non-probabilistic	Tang 2015	Blank	Perceived	Global	-0,370	-0,535	-0,178	0,000
Non-probabilistic	Thielemann 2001	Blank	Perceived	Global	-0,530	-0,632	-0,410	0,000
Non-probabilistic	Verez Cotelo 2015	Blank	Perceived	Global	-0,440	-0,711	-0,054	0,027
Non-probabilistic	Yen 2006	Blank	Perceived	Global	-0,490	-0,668	-0,258	0,000
Non-probabilistic					-0,357	-0,404	-0,309	0,000
Probabilistic	Del-Pino-Casado 2022	Blank	Perceived	Global	-0,340	-0,520	-0,131	0,002
Probabilistic	Hobbs 1997	Blank	Perceived	Global	-0,331	-0,495	-0,144	0,001
Probabilistic	Lee 2003	Blank	Perceived	Global	-0,360	-0,550	-0,135	0,002
Probabilistic	Li 2019	Blank	Perceived	Global	-0,190	-0,269	-0,109	0,000
Probabilistic	Moral Serrano 2003	Blank	Perceived	Global	-0,170	-0,297	-0,037	0,012
Probabilistic	Rauktis 1995	Blank	Perceived	Global	-0,300	-0,464	-0,116	0,002
Probabilistic	Serrani 2014	Blank	Perceived	Global	-0,217	-0,397	-0,021	0,030
Probabilistic	Serrano-Ortega 2018	Blank	Perceived	Global	-0,320	-0,446	-0,181	0,000
Probabilistic	Yun 2023	Blank	Perceived	Global	-0,340	-0,424	-0,250	0,000
Probabilistic	Zhong 2020	Blank	Perceived	Global	-0,399	-0,466	-0,327	0,000
Probabilistic					-0,296	-0,356	-0,232	0,000

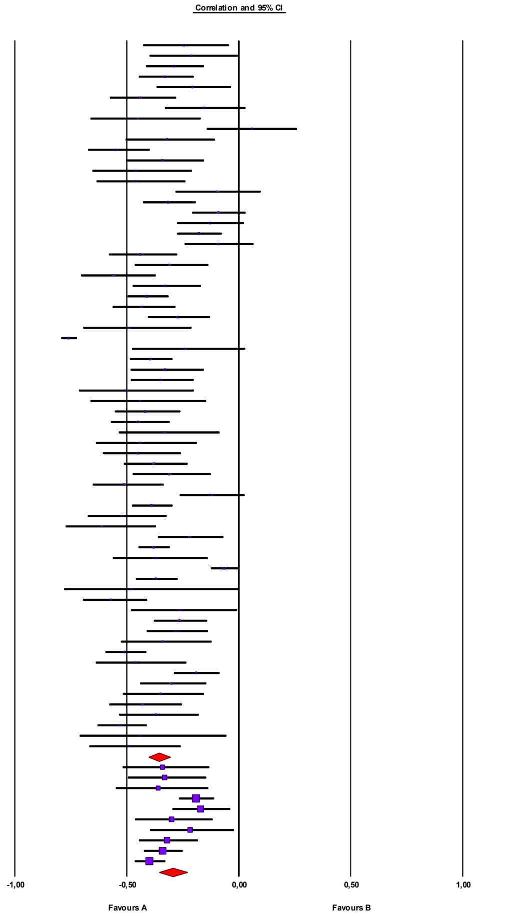

Forest plot for subgroups by control of confounders

Group by Control of confounders	Study name	Subgroup within study	Comparison	Outcome	Correlation	Lower limit	Upper limit	p-Value
No	Aggar 2010	Blank	Perceived	Global	-0,245	-0,427	-0,043	0,018
No	Ar 2017	Blank	Perceived	Global	-0,290	-0,415	-0,154	0,000
No	Arevalo-Flechas 2014	Blank	Perceived	Global	-0,330	-0,448	-0,201	0,000
No	Asti 2006	Combined	Perceived	Global	-0,207	-0,368	-0,034	0,019
No	Ballard 1995	Blank	Perceived	Global	-0,155	-0,330	0,030	0,100
No	Bergman 1992	Blank	Perceived	Global	0,060	-0,144	0,259	0,567
No	Bigatti 2011	Blank	Perceived	Global	-0,320	-0,506	-0,105	0,004
No	Bonsu 2019	Blank	Perceived	Global	-0,550	-0,674	-0,396	0,000
No	Burgeois 1996	Blank	Perceived	Global	-0,340	-0,503	-0,154	0,000
No	Calvete 2011	Blank	Perceived	Global	-0,315	-0,429	-0,192	0,000
No	Cardenas 2014	Blank	Perceived	Global	-0,090	-0,208	0,031	0,145
No	Chai 2018	Blank	Perceived	Global	-0,129	-0,276	0,024	0,099
No	Chou 2010	Blank	Perceived	Global	-0,178	-0,276	-0,075	0,001
No	Chow 2012	Blank	Perceived	Global	-0,090	-0,243	0,067	0,261
No	Crespo 2005	Blank	Perceived	Global	-0,440	-0,581	-0,274	0,000
No	Cumming 2008	Blank	Perceived	Global	-0,310	-0,466	-0,135	0,001
No	Decker 1989	Blank	Perceived	Global	-0,560	-0,705	-0,369	0,000
No	Durkin 2010	Blank	Perceived	Global	-0,330	-0,475	-0,167	0,000
No	Faber 2005	Blank	Perceived	Global	-0,410	-0,499	-0,313	0,000
No	Giovannetti 2015	Blank	Perceived	Global	-0,434	-0,565	-0,282	0,000
No	Grant 2000	Blank	Perceived	Global	-0,273	-0,407	-0,128	0,000
No	Grant 2001	Blank	Perceived	Global	-0,490	-0,695	-0,211	0,001
No	Haley 1987	Blank	Perceived	Global	-0,240	-0,477	0,030	0,080
No	Han 2014	Blank	Perceived	Global	-0,395	-0,486	-0,295	0,000
No	Hasson-Ohayon 2010	Blank	Perceived	Global	-0,350	-0,483	-0,201	0,000
No	Hobbs 1997	Blank	Perceived	Global	-0,331	-0,495	-0,144	0,001
No	Hwang 2011	Blank	Perceived	Global	-0,500	-0,714	-0,200	0,002
No	Jeong 2017	Blank	Perceived	Global	-0,440	-0,663	-0,145	0,005
No	Khusaifan 2017	Blank	Perceived	Global	-0,418	-0,555	-0,260	0,000
No	Kiral 2017	Blank	Perceived	Global	-0,450	-0,573	-0,308	0,000
No	Kusku 2009	Blank	Perceived	Global	-0,440	-0,638	-0,187	0,001
No	Leibach 2013	Blank	Perceived	Global	-0,450	-0,609	-0,257	0,000
No	Luchsinger 2015	Blank	Perceived	Global	-0,380	-0,514	-0,228	0,000
No	Majerovitz 2007	Blank	Perceived	Global	-0,310	-0,475	-0,124	0,001
No	Manso Martinez 2013	Combined	Perceived	Global	-0,511	-0,653	-0,335	0,000
No	Moral Serrano 2003	Blank	Perceived	Global	-0,170	-0,297	-0,037	0,012
No	Neri 2012	Blank	Perceived	Global	-0,122	-0,265	0,026	0,107
No	Nuwamanya 2023	Blank	Perceived	Global	-0,390	-0,477	-0,295	0,000
No	Pagel 1987	Blank	Perceived	Global	-0,520	-0,675	-0,321	0,000
No	Pearce 2006	Blank	Perceived	Global	-0,220	-0,362	-0,068	0,005
No	Raad 2020	Blank	Perceived	Global	-0,380	-0,449	-0,307	0,000
No	Rapp 1998	Blank	Perceived	Global	-0,370	-0,563	-0,139	0,002
No	Rauktis 1995	Blank	Perceived	Global	-0,300	-0,464	-0,116	0,002
No	Rivera 1991	Blank	Perceived	Global	-0,065	-0,126	-0,004	0,038
No	Robinson 1994	Blank	Perceived	Global	-0,480	-0,781	0,001	0,050
No	Rodi 2015	Blank	Perceived	Global	-0,570	-0,697	-0,408	0,000
No	Sahin 2012	Blank	Perceived	Global	-0,260	-0,482	-0,007	0,045
No	Sandoval 2019	Blank	Perceived	Global	-0,264	-0,380	-0,140	0,000
No	Schulz 1991	Blank	Perceived	Global	-0,280	-0,412	-0,136	0,000
No	Schumacher 1993	Blank	Perceived	Global	-0,340	-0,528	-0,121	0,003
No	Serrani 2014	Blank	Perceived	Global	-0,217	-0,397	-0,021	0,030
No	Shaughnessy 2011	Blank	Perceived	Global	-0,460	-0,639	-0,233	0,000
No	Shukri 2020	Blank	Perceived	Global	-0,190	-0,290	-0,085	0,000
No	Steffen 2002	Blank	Perceived	Global	-0,300	-0,441	-0,144	0,000
No	Tai 2021	Blank	Perceived	Global	-0,430	-0,579	-0,253	0,000
No	Verez Cotelo 2015	Blank	Perceived	Global	-0,440	-0,711	-0,054	0,027
No	Yen 2006	Blank	Perceived	Global	-0,490	-0,668	-0,258	0,000
No	Zhong 2020	Blank	Perceived	Global	-0,399	-0,466	-0,327	0,000
No					-0,293	-0,311	-0,274	0,000
Yes	Ali 2016	Blank	Perceived	Global	-0,210	-0,400	-0,003	0,047
Yes	Azevedo 2017	Blank	Perceived	Global	-0,439	-0,576	-0,278	0,000
Yes	Bambara 2014	Blank	Perceived	Global	-0,450	-0,663	-0,169	0,002
Yes	Burton 2008	Blank	Perceived	Global	-0,460	-0,655	-0,208	0,001
Yes	Butler 2001	Blank	Perceived	Global	-0,460	-0,637	-0,238	0,000
Yes	Cabral 2014	Blank	Perceived	Global	-0,096	-0,283	0,098	0,333
Yes	Del-Pino-Casado 2022	Blank	Perceived	Global	-0,340	-0,520	-0,131	0,002
Yes	Graven 2020	Blank	Perceived	Global	-0,760	-0,794	-0,722	0,000
Yes	Harwood 2000	Blank	Perceived	Global	-0,330	-0,485	-0,156	0,000
Yes	Koerner 2010	Blank	Perceived	Global	-0,330	-0,537	-0,085	0,009
Yes	Lee 2003	Blank	Perceived	Global	-0,360	-0,550	-0,135	0,002
Yes	Li 2019	Blank	Perceived	Global	-0,190	-0,269	-0,109	0,000
Yes	Pagnini 2010	Blank	Perceived	Global	-0,610	-0,774	-0,369	0,000
Yes	Rivera-Navarro 2018	Blank	Perceived	Global	-0,370	-0,460	-0,272	0,000
Yes	Scicolone 2018	Blank	Perceived	Global	-0,510	-0,596	-0,412	0,000
Yes	Serrano-Ortega 2018	Blank	Perceived	Global	-0,320	-0,446	-0,181	0,000
Yes	Stevens 2013	Blank	Perceived	Global	-0,350	-0,519	-0,154	0,001
Yes	Tang 2015	Blank	Perceived	Global	-0,370	-0,535	-0,178	0,000
Yes	Thielemann 2001	Blank	Perceived	Global	-0,530	-0,632	-0,410	0,000
Yes	Yun 2023	Blank	Perceived	Global	-0,340	-0,424	-0,250	0,000
Yes					-0,431	-0,458	-0,403	0,000

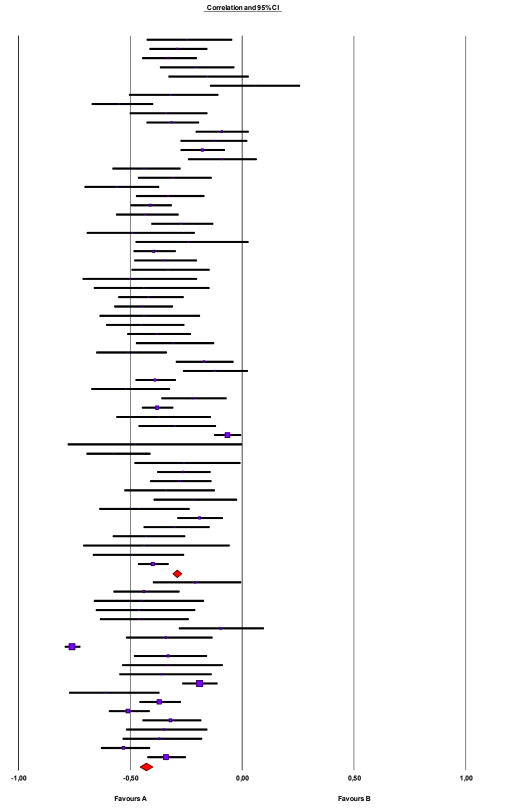

Forest plot for subgroups by care recipient

Group by Care recipients	Study name	Subgroup within study	Comparison	Outcome	Correlation	Lower limit	Upper limit	p-Value
Cancer	Bigatti 2011	Blank	Perceived	Global	-0,320	-0,506	-0,105	0,004
Cancer	Faber 2005	Blank	Perceived	Global	-0,410	-0,499	-0,313	0,000
Cancer	Han 2014	Blank	Perceived	Global	-0,395	-0,486	-0,295	0,000
Cancer	Hasson-Ohayon 2010	Blank	Perceived	Global	-0,350	-0,483	-0,201	0,000
Cancer	Jeong 2017	Blank	Perceived	Global	-0,440	-0,663	-0,145	0,005
Cancer	Kusku 2009	Blank	Perceived	Global	-0,440	-0,638	-0,187	0,001
Cancer	Nuwamanya 2023	Blank	Perceived	Global	-0,390	-0,477	-0,295	0,000
Cancer	Pearce 2006	Blank	Perceived	Global	-0,220	-0,362	-0,068	0,005
Cancer	Rodi 2015	Blank	Perceived	Global	-0,570	-0,697	-0,408	0,000
Cancer	Sahin 2012	Blank	Perceived	Global	-0,260	-0,482	-0,007	0,045
Cancer	Schumacher 1993	Blank	Perceived	Global	-0,340	-0,528	-0,121	0,003
Cancer	Shukri 2020	Blank	Perceived	Global	-0,190	-0,290	-0,085	0,000
Cancer	Thielemann 2001	Blank	Perceived	Global	-0,530	-0,632	-0,410	0,000
Cancer					-0,373	-0,435	-0,306	0,000
Dementia	Ar 2017	Blank	Perceived	Global	-0,290	-0,415	-0,154	0,000
Dementia	Arevalo-Flechas 2014	Blank	Perceived	Global	-0,330	-0,448	-0,201	0,000
Dementia	Ballard 1995	Blank	Perceived	Global	-0,155	-0,330	0,030	0,100
Dementia	Bergman 1992	Blank	Perceived	Global	0,060	-0,144	0,259	0,567
Dementia	Burgeois 1996	Blank	Perceived	Global	-0,340	-0,503	-0,154	0,000
Dementia	Cardenas 2014	Blank	Perceived	Global	-0,090	-0,208	0,031	0,145
Dementia	Haley 1987	Blank	Perceived	Global	-0,240	-0,477	0,030	0,080
Dementia	Harwood 2000	Blank	Perceived	Global	-0,330	-0,485	-0,156	0,000
Dementia	Khusaifan 2017	Blank	Perceived	Global	-0,418	-0,555	-0,260	0,000
Dementia	Kiral 2017	Blank	Perceived	Global	-0,450	-0,573	-0,308	0,000
Dementia	Lee 2003	Blank	Perceived	Global	-0,360	-0,550	-0,135	0,002
Dementia	Luchsinger 2015	Blank	Perceived	Global	-0,380	-0,514	-0,228	0,000
Dementia	Pagel 1987	Blank	Perceived	Global	-0,520	-0,675	-0,321	0,000
Dementia	Rapp 1998	Blank	Perceived	Global	-0,370	-0,563	-0,139	0,002
Dementia	Rivera-Navarro 2018	Blank	Perceived	Global	-0,370	-0,460	-0,272	0,000
Dementia	Robinson 1994	Blank	Perceived	Global	-0,480	-0,781	0,001	0,050
Dementia	Schulz 1991	Blank	Perceived	Global	-0,280	-0,412	-0,136	0,000
Dementia	Steffen 2002	Blank	Perceived	Global	-0,300	-0,441	-0,144	0,000
Dementia	Tang 2015	Blank	Perceived	Global	-0,370	-0,535	-0,178	0,000
Dementia	Verez Cotelo 2015	Blank	Perceived	Global	-0,440	-0,711	-0,054	0,027
Dementia					-0,314	-0,370	-0,255	0,000
Frail older people	Aggar 2010	Blank	Perceived	Global	-0,245	-0,427	-0,043	0,018
Frail older people	Butler 2001	Blank	Perceived	Global	-0,460	-0,637	-0,238	0,000
Frail older people	Chow 2012	Blank	Perceived	Global	-0,090	-0,243	0,067	0,261
Frail older people	Crespo 2005	Blank	Perceived	Global	-0,440	-0,581	-0,274	0,000
Frail older people	Del-Pino-Casado 2022	Blank	Perceived	Global	-0,340	-0,520	-0,131	0,002
Frail older people	Durkin 2010	Blank	Perceived	Global	-0,330	-0,475	-0,167	0,000
Frail older people	Koerner 2010	Blank	Perceived	Global	-0,330	-0,537	-0,085	0,009
Frail older people	Li 2019	Blank	Perceived	Global	-0,190	-0,269	-0,109	0,000
Frail older people	Manso Martinez 2013	Combined	Perceived	Global	-0,511	-0,653	-0,335	0,000
Frail older people	Neri 2012	Blank	Perceived	Global	-0,122	-0,265	0,026	0,107
Frail older people	Rivera 1991	Blank	Perceived	Global	-0,065	-0,126	-0,004	0,038
Frail older people	Sandoval 2019	Blank	Perceived	Global	-0,264	-0,380	-0,140	0,000
Frail older people	Scicolone 2018	Blank	Perceived	Global	-0,510	-0,596	-0,412	0,000
Frail older people	Serrani 2014	Blank	Perceived	Global	-0,217	-0,397	-0,021	0,030
Frail older people	Serrano-Ortega 2018	Blank	Perceived	Global	-0,320	-0,446	-0,181	0,000
Frail older people	Yun 2023	Blank	Perceived	Global	-0,340	-0,424	-0,250	0,000
Frail older people	Zhong 2020	Blank	Perceived	Global	-0,399	-0,466	-0,327	0,000
Frail older people					-0,303	-0,378	-0,225	0,000
Mental illness	Cabral 2014	Blank	Perceived	Global	-0,096	-0,283	0,098	0,333
Mental illness	Chai 2018	Blank	Perceived	Global	-0,129	-0,276	0,024	0,099
Mental illness	Hobbs 1997	Blank	Perceived	Global	-0,331	-0,495	-0,144	0,001
Mental illness	Rauktis 1995	Blank	Perceived	Global	-0,300	-0,464	-0,116	0,002
Mental illness	Yen 2006	Blank	Perceived	Global	-0,490	-0,668	-0,258	0,000
Mental illness					-0,258	-0,384	-0,122	0,000
Stroke	Ali 2016	Blank	Perceived	Global	-0,210	-0,400	-0,003	0,047
Stroke	Cumming 2008	Blank	Perceived	Global	-0,310	-0,466	-0,135	0,001
Stroke	Grant 2000	Blank	Perceived	Global	-0,273	-0,407	-0,128	0,000
Stroke	Grant 2001	Blank	Perceived	Global	-0,490	-0,695	-0,211	0,001
Stroke					-0,292	-0,378	-0,200	0,000

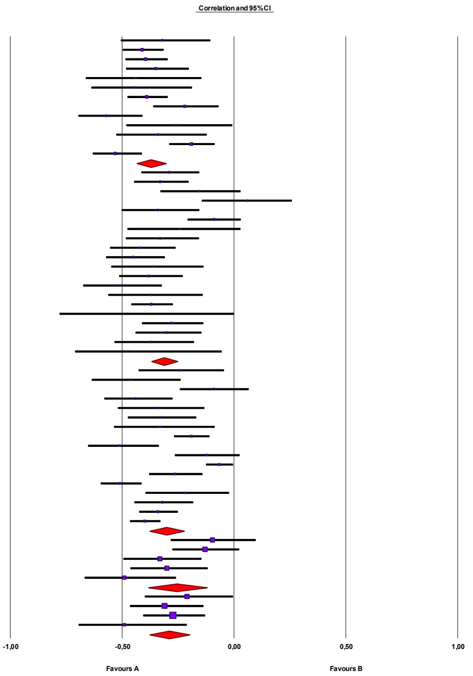

Supplement: Supplementary file 1 [file jcm-12-06468-s001.zip › FIGURE S1.pdf]
