# Supplementary material for: Association between Social Support and Depressive Symptoms in Informal Caregivers of Adult and Older Dependents: A Systematic Review and Meta-Analysis
Source: J Clin Med. 2023 Oct 11;12(20):6468. doi: 10.3390/jcm12206468 (PMC10607501; doi:10.3390/jcm12206468)

SCATTERPLOTS FOR META-REGRESSIONS

Scatterplots for perceived social support

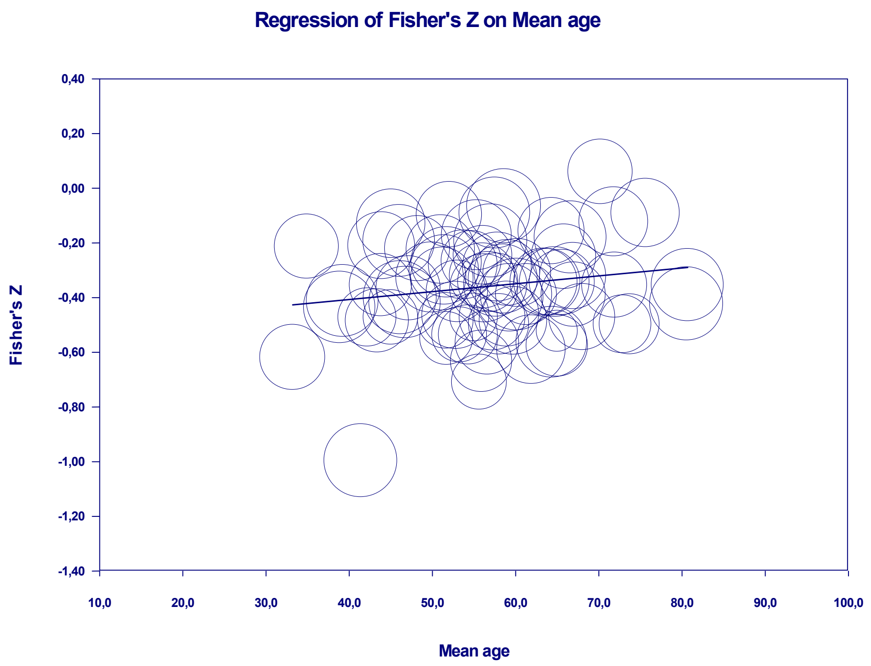

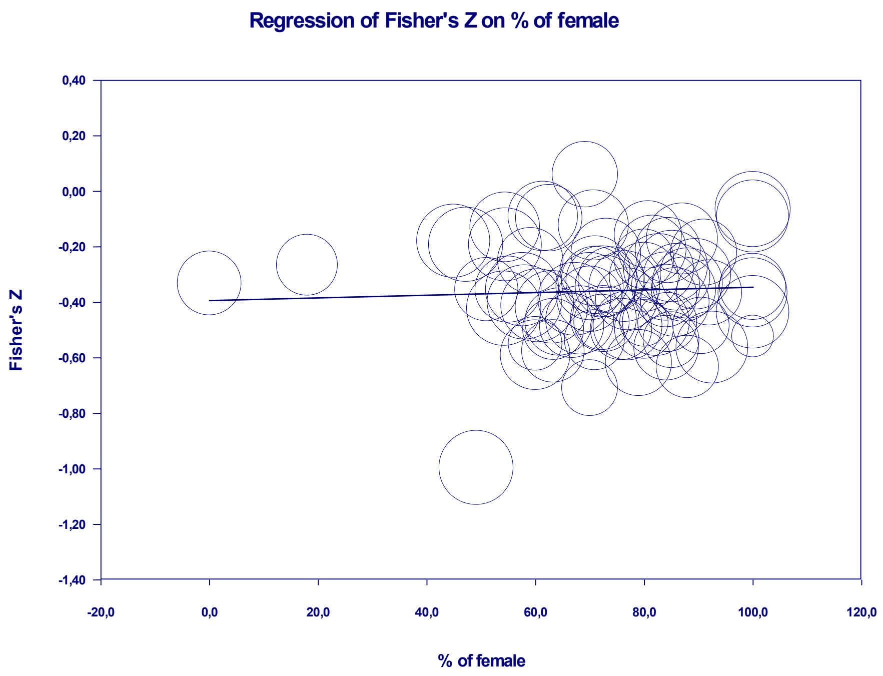

Scatterplots for received social support

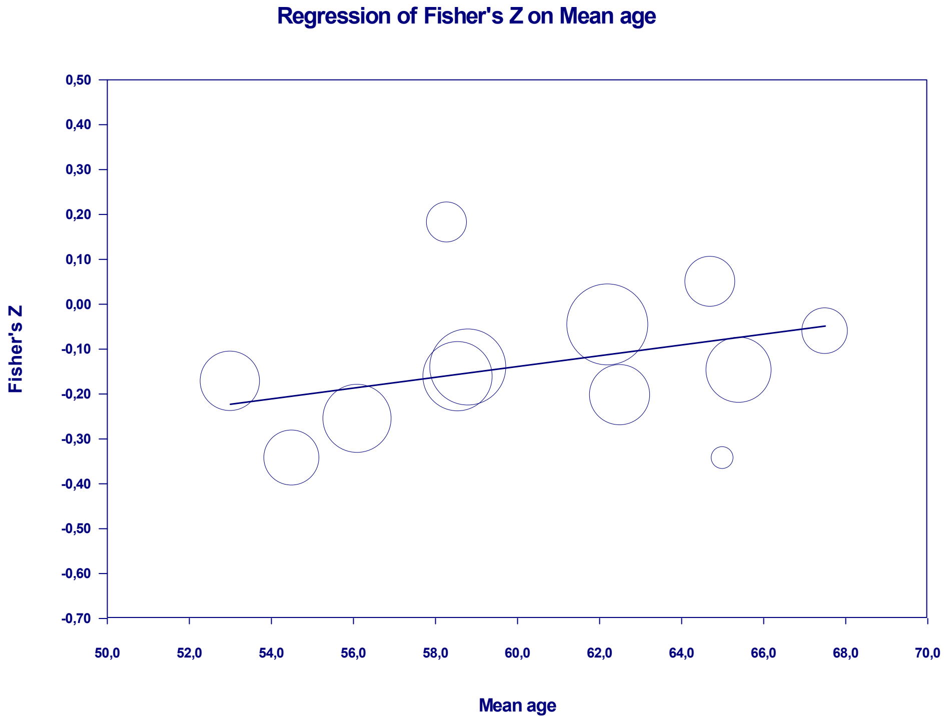

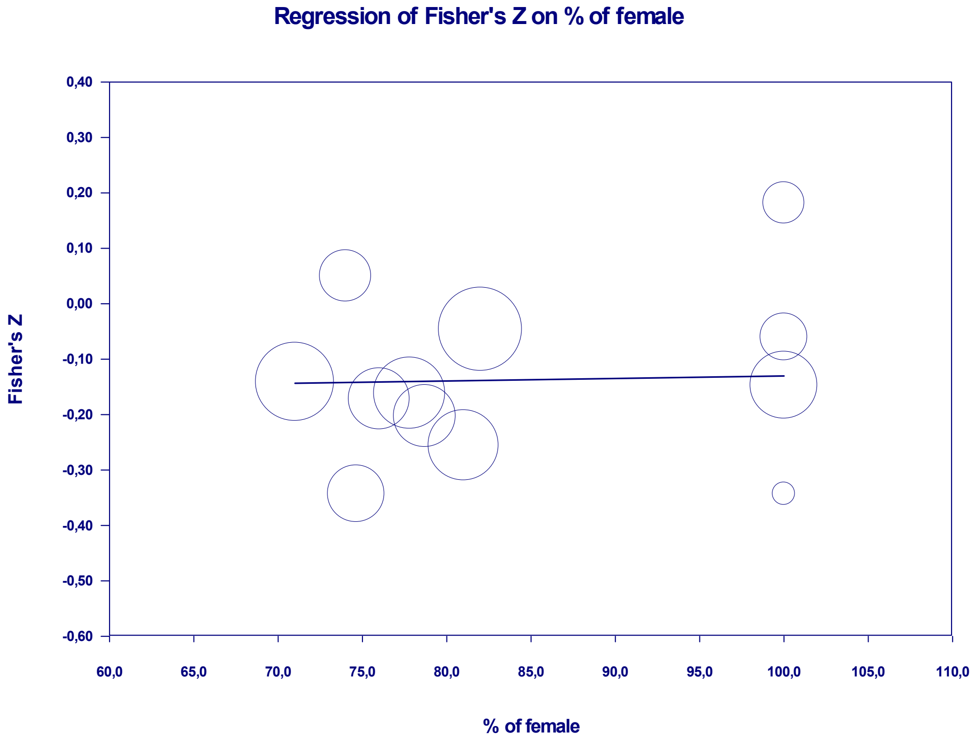

Supplement: Supplementary file 1 [file jcm-12-06468-s001.zip › FIGURE S2.pdf]
