# Supplementary material for: Association between Social Support and Depressive Symptoms in Informal Caregivers of Adult and Older Dependents: A Systematic Review and Meta-Analysis
Source: J Clin Med. 2023 Oct 11;12(20):6468. doi: 10.3390/jcm12206468 (PMC10607501; doi:10.3390/jcm12206468)
Supplement: Supplementary file 1 [file jcm-12-06468-s001.zip › TABLE S1 Summary of the search strategy used in the databases.pdf]

Table S1: Summary of the search strategy used in the databases

Database	Search string	Date	Sample
Pubmed	((Depression[mj] OR ((Depress*[tiab]) NOT MEDLINE[sb])) AND (Caregivers[mj] OR ((Caregiv*[tiab] OR Care giv*[tiab] OR Carer*[tiab]) NOT MEDLINE[sb])) AND (Social support[mh:noexp] OR ((social network[tiab] OR informal support[tiab] OR received support[tiab] OR perceived support[tiab]) NOT MEDLINE[sb]))) NOT (clinical trial[pt] OR randomized clinical trial[tiab] OR randomized controlled trial[tiab] OR qualitative study OR qualitative research)	15 January 2023	241
CINAHL	(MJ Depression OR Depress*) AND (MJ Caregivers OR AB Caregiv* OR AB Care giv* OR AB Carer*) AND (MJ social support OR AB social network OR AB informal support OR AB received support OR AB perceived support) NOT (PT clinical trial OR AB randomized clinical trial OR randomized controlled trial OR RCT OR qualitative study OR qualitative research)	15 January 2023	394
PsyInfo	(MJSUB(Depression) AND MJSUB(Caregivers) AND MJSUB(social support)) NOT (PT(clinical trial) OR AB("randomized clinical trial") OR AB("randomized controlled trial") OR AB(RCT) OR AB("Qualitative study") OR AB("qualitative research") OR AB("systematic review") OR AB("experimental research") OR TI("Qualitative study") OR TI("qualitative research") OR TI("systematic review") OR TI("experimental research") OR SU(support groups))	15 January 2023	351
Scopus	Depression(mj) AND Caregivers(mj) AND ("social support" OR "social network" OR "informal support" OR "received support" OR "perceived support")	15 January 2023	2,495
